# High-Flow Nasal Cannula Versus Noninvasive Ventilation for Prevention of Reintubation in High-Risk Critically Ill Patients (HIGH-FLOW OXY): An Informative Systematic Review and Meta-Analysis

**DOI:** 10.1097/CCE.0000000000001452

**Published:** 2026-08-06

**Authors:** Nhan Nguyen, Nathalia Alves de Barros e Lyra, David Downes, Nghi Bao Tran, Vinh Quang Tri Ho, Yacin Zawam, Vy Ngoc Dan Nguyen, Ha Duc Thien Le, Jafar Aljazeeri

**Affiliations:** 1 Faculty of Medicine, Department of Internal Medicine, University of Debrecen, Debrecen, Hungary.; 2 Department of Pediatrics, SUNY Upstate University, Syracuse, NY.; 3 Faculdade Pernambucana de Saude, Recife, Pernambuco, Brazil.; 4 Department of Rural Medicine, The University of New England, Armidale, NSW, Australia.; 5 Southern Ohio Medical Center, Portsmouth, OH.; 6 Department of Nursing, The University of Melbourne, Melbourne, VIC, Australia.; 7 University of Pittsburgh Medical Center, Harrisburg, PA.; 8 Drexel University College of Medicine, Philadelphia, PA.; 9 Medical City Hospital for Military and Security Services, Muscat, Oman.

**Keywords:** critical illness, high-flow nasal cannula, high risk of reintubation, noninvasive ventilation, very high risk

## Abstract

**OBJECTIVES:**

Optimal postextubation respiratory support for critically ill patients at high risk of reintubation remains uncertain. We aimed to compare the efficacy of high-flow nasal cannula (HFNC) vs. noninvasive ventilation (NIV) in preventing reintubation among critically ill adults at high risk of extubation failure.

**DATA SOURCES:**

PubMed, Cochrane Central Register of Controlled Trials, Embase, and ClinicalTrials.gov were systematically searched from inception through the latest available date.

**STUDY SELECTION:**

HIGH-FLOW OXY is a systematic review and meta-analysis comparing HFNC with NIV in ICU patients at high risk of extubation failure. The primary outcome was short-term reintubation within 3 days postextubation. Secondary outcomes included ICU mortality, ICU length of stay, sepsis, and nosocomial pneumonia.

**DATA EXTRACTION:**

Data were extracted using predefined criteria. Risk of bias (RoB) was assessed with the Cochrane tool, and certainty of evidence was evaluated using Grading of Recommendations, Assessment, Development, and Evaluation. Random-effects models were used for pooled analyses. Prespecified subgroup analyses included obese patients, very high-risk patients, elderly individuals, and those with acute exacerbations of chronic obstructive pulmonary disease.

**DATA SYNTHESIS:**

Fifteen randomized controlled trials (RCTs; *n* = 2073) were included. HFNC and NIV did not differ significantly in reintubation (odds ratio [OR], 1.19; 95% CI, 0.88–1.59), ICU mortality (OR, 0.74; 95% CI, 0.40–1.38), sepsis (OR, 1.29; 95% CI, 0.71–2.35), nosocomial pneumonia (OR, 1.01; 95% CI, 0.67–1.52), or ICU length of stay (MD −0.37 d; 95% CI, −1.42 to 0.68). Subgroup analyses suggested a higher reintubation risk with HFNC among very high-risk patients (OR, 1.63; 95% CI, 1.05–2.53) and nonoperative obese patients (OR, 2.52; 95% CI, 1.45–4.38). Sensitivity analysis excluding trials at high RoB indicated a potential increase in reintubation with HFNC (OR, 1.32; 95% CI, 1.02–1.71). Overall, certainty of evidence was low across outcomes.

**CONCLUSIONS:**

Among ICU patients at high risk of extubation failure, HFNC did not reduce reintubation compared with NIV, and may be associated with increased risk in selected high-risk subgroups. Further adequately powered, risk-stratified RCTs are warranted to define the optimal postextubation respiratory support strategy.

**KEY POINTS**

**Question:** Does high-flow nasal cannula (HFNC) provide clinical outcomes comparable to noninvasive ventilation (NIV) in critically ill patients at high risk of reintubation?

**Findings:** In this meta-analysis, HFNC demonstrated overall outcomes comparable to NIV. However, subgroup analyses suggested heterogeneity in treatment effects. HFNC was associated with a higher rate of early reintubation among patients with four or more risk factors for extubation failure (very high risk) and among nonoperative obese patients. Conversely, HFNC was associated with a shorter ICU length of stay in patients with acute exacerbation of chronic obstructive pulmonary disease. Analyses restricted to high-quality trials suggested a potential increase in reintubation risk with HFNC in high-risk populations.

**Meaning:** Among critically ill patients at high risk of reintubation, HFNC may be associated with a higher risk of reintubation than NIV in certain subgroups. However, these findings should be considered hypothesis-generating, given substantial residual uncertainty in the current evidence and heterogeneity in study populations and treatment protocols.

In the ICU, approximately 10–15% of patients require reintubation due to postextubation respiratory failure (1, 2). Reintubation is associated with prolonged hospitalization, increased healthcare costs, and higher mortality (3, 4), making its prevention a key clinical priority. Evidence from randomized controlled trials (RCTs) (5, 6) and meta-analyses (7) indicates that high-flow nasal cannula (HFNC) may provide outcomes comparable to noninvasive ventilation (NIV) for preventing reintubation after extubation, particularly in patients at high risk of extubation failure.

HFNC delivers heated and humidified gas with an adjustable mixture of air and oxygen through a large-bore nasal cannula, whereas NIV provides ventilatory support by delivering positive airway pressure via a nasal or oronasal mask without the need for an invasive airway. Patients considered at high risk of reintubation or extubation failure have traditionally been defined as those with at least one of several clinical characteristics, including (8, 9) age older than 65 years; Acute Physiology and Chronic Health Evaluation II (APACHE II) score greater than 12 at extubation; body mass index (BMI) greater than 30 kg/m^2^; impaired secretion clearance; difficult or prolonged weaning; presence of two or more comorbidities; acute heart failure as the indication for mechanical ventilation; chronic obstructive pulmonary disease (COPD); airway patency problems; prolonged duration of mechanical ventilation; or hypercapnia at the end of the spontaneous breathing trial.

However, emerging evidence suggests that the comparative effectiveness of HFNC and NIV may differ across specific high-risk phenotypes. A post hoc analysis by Hernández et al (10) suggested that NIV may provide greater benefit in patients with four or more risk factors for extubation failure, whereas HFNC appeared comparable to NIV in obese patients at risk of reintubation (11). Despite these observations, prior meta-analyses (8) have not systematically evaluated whether the relative effects of HFNC and NIV differ across prespecified high-risk subgroups or focused on single risk factors (12, 13). However, the optimal postextubation respiratory support strategy for specific high-risk patient subgroups remains uncertain.

Therefore, we conducted the HIGH FLOW nasal OXYgen cannula vs. Noninvasive Ventilation in critically ill patients at high risk of reintubation (HIGH-FLOW OXY) meta-analysis to compare HFNC and NIV as standalone postextubation respiratory support strategies in critically ill patients at risk of reintubation. We also performed prespecified subgroup analyses based on clinical risk factors, including COPD, very high-risk status (≥4 risk factors), and obesity, to explore potential heterogeneity in treatment effects and inform future targeted clinical trials.

## MATERIALS AND METHODS

This systematic review and meta-analysis were conducted in accordance with the Cochrane Collaboration Handbook for Systematic Review of Interventions and reported following the Preferred Reporting Items for Systematic Reviews and Meta-Analysis (PRISMA) Statement guidelines (14, 15); as it used previously published data without involving human participants, institutional review board approval and informed consent were not required, and the study adhered to the ethical standards of the World Medical Association Declaration of Helsinki (1975, as revised).

### Eligibility Criteria

We included RCTs directly comparing HFNC with NIV in adults (≥18 y) admitted to the ICU who were at high risk of reintubation. Eligible studies were required to report at least one predefined clinical outcome. High risk of reintubation was defined by the presence of one or more of the following: age older than 65 years, APACHE II score greater than 12 on the day of extubation, obesity (BMI ≥ 30 kg/m^2^ or race-specific BMI cutoff where applicable), poor secretion management, difficult or prolonged weaning, two or more comorbidities, acute heart failure requiring mechanical ventilation, COPD, airway patency problems, prolonged mechanical ventilation, or hypercapnia at the end of a spontaneous breathing trial.

We excluded observational studies, single-arm studies, conference abstracts, pediatric-only populations, trials comparing NIV with conventional oxygen therapy without a separate HFNC subgroup, and studies using HFNC adjunctively with NIV. The predefined primary outcome was short-term reintubation within 72 hours of extubation. Secondary outcomes included sepsis, nosocomial pneumonia, ICU length of stay, and ICU mortality. Although duration of mechanical ventilation was prespecified, formal analysis was not feasible due to limited data (reported in only two RCTs).

Prespecified subgroup analyses of reintubation were conducted, where data allowed, to enhance clinical relevance. Subgroups included very high-risk patients (≥4 risk factors), obese patients (operative vs. nonoperative), and patients with acute exacerbation of chronic obstructive pulmonary disease (AECOPD) and hypercapnic respiratory failure. An additional subgroup restricted to high-quality studies (excluding trials at high RoB) was performed. Subgroup data were extracted from published or **Supplementary Material** (https://links.lww.com/CCX/B658). Random-effects models were applied, estimating odds ratios for dichotomous and mean differences (MDs) for continuous outcomes. Formal statistical tests for interaction between subgroups were not performed; therefore, subgroup findings should be interpreted as hypothesis-generating. Secondary outcomes were analyzed when sufficient data were available.

### Search Strategies and Data Extraction

**Figure 1.**
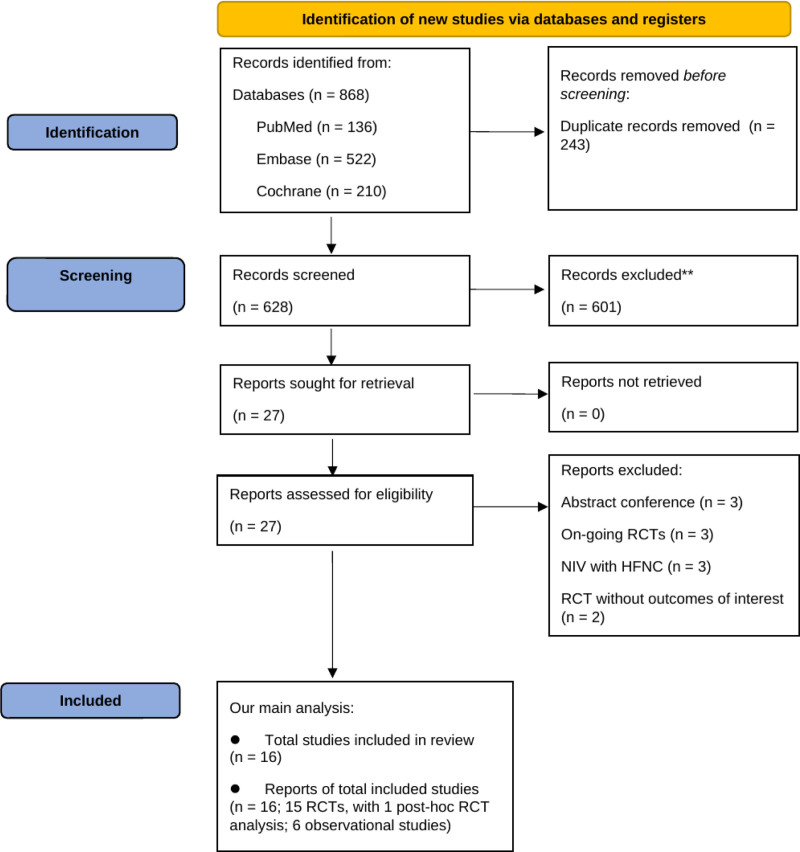
The Preferred Reporting Items for Systematic Reviews and Meta-Analyses flowchart (10) of study selection. HFNC = high-flow nasal cannula, NIV = noninvasive ventilation, RCT = randomized controlled trial.

A comprehensive search of major databases (PubMed/MEDLINE, Embase, Scopus, and Cochrane Central) was performed from inception to February 2026. The full search strategies for each database are provided in **Appendix 1** (https://links.lww.com/CCX/B669). Reference lists of relevant systematic reviews and included studies were also manually screened for additional eligible trials. Study selection was conducted in two stages: titles and abstracts were independently screened by two reviewers (N.N. and N.A.d.B.e.L.) to identify potentially eligible studies, followed by independent full-text review. Data extraction was performed independently and in duplicate by N.B.T. and Y.Z. using a standardized form, with forest plot data independently verified by H.D.T.L. and V.N.D.N. Discrepancies were resolved through discussion or, if needed, by a third reviewer (N.N.). This meta-analysis was prospectively registered on PROSPERO under ID: CRD420261289624 (registered January 20, 2026). Study selection and screening results are summarized in a PRISMA flow diagram (**Fig. 1**).

### Statistical Analysis

Two reviewers (N.B.T. and Y.Z.) independently extracted baseline characteristics (**Supplementary Table 1**, https://links.lww.com/CCX/B658) and outcome data. Dichotomous outcomes were pooled as odds ratios (ORs) with 95% CIs. Continuous outcomes were analyzed as MDs, since all studies reported results in consistent units (days), which improves clinical interpretability compared with standardized mean differences. A difference of greater than or equal to 1 day was considered the minimal clinically important difference.

Heterogeneity was assessed using the *I*^2^ statistic and Cochrane *Q* test, with *I*^2^ greater than 50% and *p* value of less than 0.10 indicating substantial heterogeneity. All meta-analyses used a random-effects model (DerSimonian and Laird) to account for within- and between-study variability. Statistical analyses were conducted in Review Manager (RevMan) version 5.4 (Cochrane Collaboration, Copenhagen, Denmark). Sensitivity analyses employed a leave-one-out approach, and subgroup analyses were performed when data allowed. Assessment of small-study effects was planned when greater than or equal to 10 studies were available for an outcome, using funnel plots and Egger’s regression test.

### Quality Assessment

Two independent reviewers (N.A.d.B.e.L and N.B.T.) assessed the RoB using the Cochrane RoB 2.0 tool (Bristol Medical School, Bristol, United Kingdom) (16), across five domains: randomization process, deviations from intended interventions, missing outcome data, measurement of outcomes, and selection of reported results. Each domain was rated as low, high, or unclear risk, with discrepancies resolved by consensus or a third reviewer (N.N.). Publication bias was evaluated using funnel plots and, if uncertain, further assessed with Egger’s test (D.D.).

The certainty of evidence was appraised using the Grading of Recommendations, Assessment, Development, and Evaluation (GRADE) (17) approach by (V.H.Q.T. and Y.Z.), considering risk of bias, imprecision, inconsistency, indirectness, and publication bias. Outcomes were rated as high, moderate, low, or very low certainty. All outcomes were evaluated to determine the overall strength and reliability of the HIGH-FLOW OXY meta-analysis findings.

## RESULTS

### Study Selection and Baseline Characteristics

The literature search identified 868 records. After removing duplicates and screening titles and abstracts, 30 studies underwent full-text review. Ten studies were excluded: two conference abstracts (18, 19), three ongoing trials (20–22), three studies that alternated NIV with HFNC (23–25), one post hoc analysis lacking standalone HFNC subgroup data (26), and two trials without relevant outcomes (27, 28) (**Supplementary Table 3**, https://links.lww.com/CCX/B658). Ultimately, 15 RCTs (5, 6, 29–40), including one post hoc analysis of Hernández et al (10), met inclusion criteria, enrolling 2073 critically ill patients with predefined extubation failure risk factors (Fig. 1).

Across the included trials, HFNC and NIV were evaluated as postextubation respiratory support strategies in critically ill patients at high risk of extubation failure.

All trials enrolled high-risk patients, with prespecified subpopulations including AECOPD with hypercapnic respiratory failure (29, 32, 33, 35, 37), very high-risk patients (≥4 risk factors) (10, 30), elderly (>65 y) (39), obese patients (11, 34, 36), patient at high risk (<4 risk factors) (5, 6), patients with APACHE II greater than or equal to 12 (31, 38), and those with greater than or equal to 2 comorbidities (34). Baseline demographic and clinical characteristics were broadly similar across treatment groups, with more than 50% of participants being male. Median APACHE II scores were greater than or equal to 10 in 10 trials, indicating moderate-to-high illness severity (Supplementary Table 1, https://links.lww.com/CCX/B658). Detailed intervention characteristics for HFNC and NIV protocols are summarized in **Supplementary Table 6**, https://links.lww.com/CCX/B658.

#### Short-Termed Reintubation, Within 72 h

**Figure 2.**
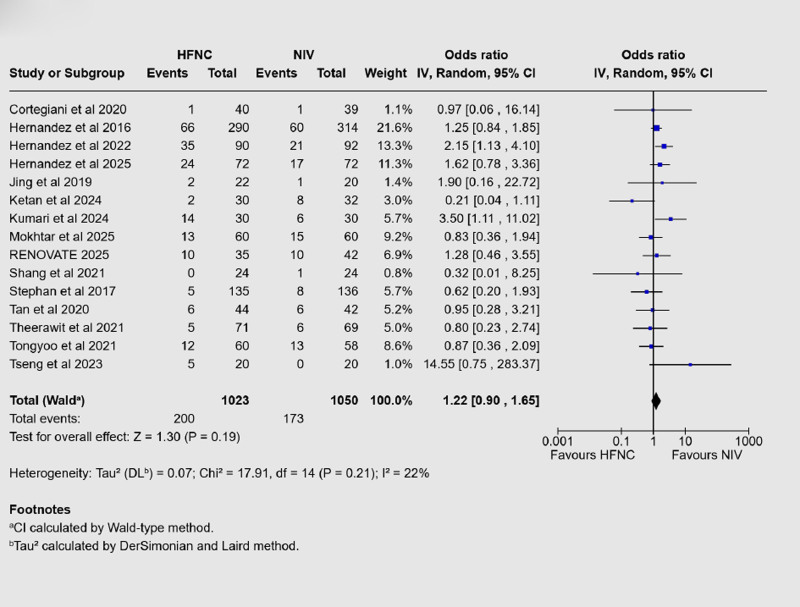
Short-term reintubation, within the first 72 h after extubation. Comparison of reintubation outcomes between high-flow nasal cannula (HFNC) and noninvasive ventilation (NIV). The figure shows odds ratios (OR) with 95% CIs. There is no statistically significant difference between the two therapies, as indicated by 95% CIs crossing 1 and *p* > 0.05.

In the HIGH-FLOW OXY meta-analysis, there were no statistically significant differences between HFNC and NIV in the risk of reintubation within 72 hours among high-risk critically ill patients (OR, 1.19; 95% CI, 0.88–1.59; low certainty of evidence; **Fig. 2**).

#### Mortality, Infection Risk, and ICU Length of Stay

Adverse events, including ICU mortality, sepsis, nosocomial pneumonia, and ICU length, did not differ significantly between HFNC and NIV: ICU mortality (OR, 0.74; 95% CI, 0.40–1.38; low certainty of evidence; **Supplementary Fig. 1**, https://links.lww.com/CCX/B658), Sepsis (OR, 1.29; 95% CI, 0.71–2.35; low certainty of evidence; **Supplementary Fig. 2** https://links.lww.com/CCX/B658), Nosocomial pneumonia (OR, 1.01; 95% CI 0.67–1.52; low certainty of evidence; **Supplementary Fig. 3**, https://links.lww.com/CCX/B658), and ICU length of stay (MD, –0.49; 95% CI, –1.38 to 0.41; very low certainty of evidence; **Supplementary Fig. 4**, https://links.lww.com/CCX/B658). However, substantial heterogeneity was observed in ICU length of stay (*I*^2^ = 89%).

### Subgroup Analyses of Primary Outcome

#### Very High Risk of Extubation Failure

This subgroup analysis included three RCTs: Hernandez et al (30), a post hoc analysis of Hernandez et al (10), and Tseng et al (40). Tseng et al (40) reported a median number of 5 (interquartile range, 4–6) risk factors for extubation failure, while both Hernandez et al focused specifically on four and above risk factors. HFNC was associated with a significantly higher risk of reintubation compared with NIV (OR, 1.63; 95% CI, 1.05–2.53; *I*^2^ = 0%; **Supplementary Fig. 5**, https://links.lww.com/CCX/B658).

#### Obesity

Four RCTs evaluated obese patients in nonoperative settings. Hernandez et al (11) specifically enrolled obese patients, while a post hoc analysis of Hernandez et al (10) reported outcomes in an obese subgroup; both trials included patients with mixed critical illnesses. Tongyoo et al (39) focused on patients with sepsis and provided a subgroup analysis of participants with BMI greater than or equal to 30 kg/m^2^ for the reintubation outcome. Kumari et al (34) was included despite mean BMIs of approximately 27–28 kg/m^2^ in the HFNC and NIV groups, as the study was conducted in India where obesity is defined as BMI greater than or equal to 25 kg/m^2^ (41). One additional RCT enrolled operative patients undergoing cardiothoracic surgery (Stephane et al [36]) and was analyzed separately.

Two meta-analyses were performed, including and excluding Kumari et al (34). When included, HFNC was associated with a higher risk of reintubation within 7 days after extubation compared with NIV in obese patients (OR, 2.11; 95% CI, 1.12–4.00; *p* = 0.02; *I*^2^ = 42%; **Supplementary Fig. 6**, https://links.lww.com/CCX/B658). Restricting the analysis to nonoperative patients yielded similar results with no observed heterogeneity (OR, 2.68; 95% CI, 1.63–4.41; *p* = 0.0001; *I*^2^ = 0%; **Supplementary Fig. 7**, https://links.lww.com/CCX/B658). Excluding Kumari et al (34), the overall obese analysis was not statistically significant and showed moderate heterogeneity (*p* = 0.11; *I*^2^ = 50%). However, in the nonoperative obese subgroup, HFNC use was associated with a two-fold increase in the risk of reintubation with minimal heterogeneity (OR, 2.52; 95% CI, 1.45–4.38; *p* = 0.001; *I*^2^ = 0%); **Supplementary Figures 8** and **9** (https://links.lww.com/CCX/B658).

#### Acute Exacerbation of COPD With Hypercapnic Respiratory Failure

During our selection process, we identified five studies (29, 32, 33, 35, 37) focusing on acute exacerbation of COPD. Overall, the analysis showed no significant difference in reintubation within 72 hours between HFNC and NIV (OR, 0.69; 95% CI, 0.29–1.63; *p* = 0.40; *I*^2^ = 0%; Supplementary Fig. 10, https://links.lww.com/CCX/B658).

#### High-Quality Trials

In this subgroup, only trials without a high risk of bias according to the Cochrane RoB 2 tool were included, excluding three high-risk RCTs (6, 31, 33). After exclusion, the analysis showed a significantly higher risk of reintubation with HFNC compared with NIV in high-risk patients (OR, 1.32; 95% CI, 1.02–1.71; *p* = 0.04; *I*^2^ = 0%). In contrast, patients with fewer than four risk factors showed no difference between HFNC and NIV (OR, 1.05; 95% CI, 0.71–1.57; *p* = 0.79; *I*^2^ = 21%). These results suggest that the signal of harm with HFNC may be driven by very high-risk patients, supporting a phenotype-specific treatment effect, **Supplementary Figures 11** and **12** (https://links.lww.com/CCX/B658).

### Quality Assessment

#### Risk of Bias

Using the RoB 2 tool (42), most trials included in HIGH-FLOW OXY were judged to have *some concerns* regarding overall risk of bias. While objective outcomes such as mortality and reintubation mitigate the impact of performance and detection bias, all trials raised concerns about deviations from intended interventions, primarily due to open-label designs (**Supplementary Table 2**, https://links.lww.com/CCX/B658). Three trials—Mokhtar et al (6), Shang et al (31) and Ketan et al (33)—were rated as *high risk* of bias owing to inadequate reporting of the randomization process, with randomization stated but not described. All included observational studies were assessed as having a high risk of bias. Egger’s test (*p* > 0.05) and symmetric funnel plots did not suggest publication bias.

#### GRADE Assessment

None of the primary outcomes were rated as high certainty despite no evidence of publication bias (**Supplementary Figs. 17–26**, https://links.lww.com/CCX/B658). All were downgraded to low certainty due to risk of bias (mainly due to deviations from intended interventions and high risk of bias arising from the randomization process in three studies) and imprecision. In subgroup analysis for reintubation, high certainty was observed only in the very high-risk group (≥4 risk factors). Moderate certainty was found in the nonoperative obese group (excluding Kumari et al [34]) and in high-quality studies, whereas AECOPD and lower-risk (<4 factors) groups remained low certainty. Excluding high-risk studies did not improve secondary outcome certainty due to persistent imprecision and overall “some concerns” bias (**Supplementary Table 5**, https://links.lww.com/CCX/B658).

#### Sensitivity Analysis

We assessed whether methodological differences in three high-risk trials (6, 31, 33) explained heterogeneity in secondary outcomes and ICU length of stay. Excluding these studies did not change the overall results, with no significant differences between HFNC and NIV in ICU length of stay, sepsis, or nosocomial pneumonia (all *p* > 0.05). For ICU length of stay outcome, HFNC was associated with shorter stay in patients with acute COPD exacerbations (MD, −1.04 d; *p* < 0.0001) and in those with fewer than four general risk factors (MD, −1.13 d; *p* < 0.0001). In contrast, HFNC was linked to longer stay in very high-risk patients (MD, 3.12 d; *p* < 0.00001), while lengths of stay were comparable in obese nonoperative patients and those with high APACHE II scores (*p* = 0.06 and *p* = 0.52; **Supplementary Figs. 12–17**, https://links.lww.com/CCX/B658).

## DISCUSSION

This meta-analysis (HIGH-FLOW OXY), including 15 RCTs (5, 6, 29–40) comprising 2073 critically ill patients with at least one risk factor for reintubation, found no significant differences between HFNC and NIV across all evaluated outcomes, including mortality, sepsis, nosocomial pneumonia, ICU length of stay, and the primary outcome of reintubation within 72 hours. These findings suggest that, overall, HFNC and NIV provide comparable clinical outcomes in heterogeneous populations of critically ill patients at increased risk of extubation failure (**Table 1**).

**TABLE 1.** Summary of Main Findings with Grading of Recommendations, Assessment, Development, and Evaluation

Outcomes	Number of Randomized Controlled Trials	High-Flow Nasal cannula, *n*	Noninvasive Ventilation, *n*	Estimate Effect (OR/MD; 95% CI)	GRADE results^a^
Reintubation, 72 hr	14	988	1008	OR, 1.19; 95% CI, 0.88–1.59	Low certainty
Sepsis	4	512	538	OR 1.29; 95% CI 0.71–2.35	Low certainty
Nosocomial pneumonia	5	647	674	OR 1.01; 95% CI 0.67–1.52	Low certainty
ICU length of stay	9	654	683	MD –0.49; 95% CI –1.38–0.41	Very Low certainty
ICU mortality	5	496	522	OR 0.74; 95% CI 0.40–1.38	Low certainty
Subgroup analysis (reintubation)					
Very high risk, more than four risk factors	3	224	206	OR 1.63; 95% CI, 1.05–2.53	High certainty
Nonoperative obese	3	158	161	OR 2.52; 95% CI, 1.45–4.38	Moderate certainty
Acute exacerbation of Chronic Obstructive Pulmonary Disease with hypercapnic respiratory failure	4	135	134	OR 0.69; 95% CI, 0.29–1.63	Low certainty

GRADE = Grading of Recommendations, Assessment, Development, and Evaluation, MD = mean difference, *n* = number of patients/events, OR = odds ratio.

a The certainty of evidence was appraised using the GRADE.

Subgroup analyses of RCTs focusing on reintubation revealed important heterogeneity. HFNC was associated with a higher reintubation risk among patients at very high risk (≥4 risk factors) and among nonoperative obese patients. Because subgroup analyses were based on aggregated trial-level data rather than individual patient data, potential ecological bias cannot be excluded, and the observed phenotype-specific signals should be interpreted cautiously. In contrast, reintubation rates were comparable between HFNC and NIV in patients with hypercapnic AECOPD (Table 1).

The increased reintubation risk observed in patients with four or more risk factors, but not in those with fewer risk factors, likely reflects cumulative risk effects, consistent with prior reports by Casey et al (43) and Hernández et al (30). These findings support the concept that the effectiveness of postextubation respiratory support may vary according to baseline risk burden. Although obesity as a general subgroup showed comparable outcomes between HFNC and NIV, persistent heterogeneity may be attributable to inclusion of postoperative patients (36), consistent with a previous network meta-analysis (12). When analyses were restricted to nonoperative critically ill patients, a signal of harm with HFNC emerged, potentially due to insufficient positive airway pressure to counter obesity-related alveolar collapse (44) and improve alveolar ventilation (45). These findings are aligned with Hernández et al (11), who suggested a trend toward NIV benefit in preventing reintubation within 7 days postextubation. Although the HIGH-WEAN trial (Thille et al [25]) was excluded due to its combined HFNC-NIV intervention, both its results and those of the present analysis support the hypothesis that alternating strategies may be beneficial for selected high-risk patients.

Patients with hypercapnic respiratory failure due to AECOPD were classified as high risk for reintubation, given their elevated vulnerability within the COPD population (46). Subgroup analysis showed comparable reintubation rates between HFNC and NIV, consistent with previous trials (28, 29, 32, 33, 35, 37) and a prior meta-analysis (47). In this phenotype, both oxygen delivery and positive pressure support appear to play central roles in preventing postextubation respiratory failure (48).

For ICU length of stay, substantial heterogeneity was observed (*I*^2^ = 89%). Given this high heterogeneity and wide CIs, pooled estimates should be interpreted cautiously. Sensitivity analyses suggested a possible modest reduction in ICU length of stay (MD of 24 hr) with HFNC in patients with AECOPD populations, but these findings remain uncertain given high rates of treatment failure and crossover to NIV reported in the previous meta-analysis (47), which included similar RCTs to those analyzed in the present study. Conversely, HFNC was associated with numerically longer ICU stays among very high-risk patients (≥4 risk factors), while data were insufficient to draw firm conclusions for other subgroups defined by lower numbers of risk factors.

Furthermore, in the overall cohort of high-risk critically ill patients, HFNC was not associated with a statistically significant difference in reintubation compared with NIV (19.6% vs. 16.5%; OR, 1.19; 95% CI, 0.88–1.59), with the CI encompassing both potential benefit and harm. Based on these event rates and assuming a two-sided α of 0.05 and 80% power, approximately 2400 patients per group (4830 total) would be required to detect a difference of this magnitude using standard sample size estimation methods (49). This highlights that trials in unselected high-risk populations would be large and resource-intensive. In contrast, analyses of selected high-risk subgroups may allow more feasible trial designs, although these subgroup findings warrant confirmation in future studies. Among patients with four or more risk factors, HFNC was associated with a higher risk of reintubation (32.1% vs. 21.8%), requiring approximately 300 patients per group, while in nonoperative obese patients, the two-fold increased risk (31.0% vs. 14.9%) requires around 100 patients per group. Focusing on high-risk subgroups enhances statistical efficiency, improves detection of meaningful differences, and provides guidance for future critical care trials.

In the GRADE assessment, certainty was low for all pooled outcomes due to risk of bias from deviations from intended interventions and wide CIs. ICU length of stay was further downgraded to very low certainty because of substantial heterogeneity. Therefore, our confidence in the effect estimates is limited, and the true effect may differ substantially from the estimated effect. Importantly, subgroup analyses were not powered for definitive inference, and findings should therefore be interpreted cautiously. Additionally, multiple subgroup analyses increase the probability of spurious findings due to multiplicity, and therefore these results should be interpreted as exploratory rather than confirmatory. Overall, these findings highlight the considerable uncertainty surrounding the potential increased reintubation risk associated with HFNC in certain subgroups, and the need for phenotype-specific randomized trials to better identify patients most likely to benefit from HFNC.

### Limitation

Although HIGH-FLOW OXY offers valuable insights into the heterogeneity of HFNC effects among patients at high risk of reintubation using both frequentist and Bayesian methods, several limitations exist. Data were limited for key subgroups, especially elderly patients; although many trials reported mean ages above 65 years, dedicated subgroup analyses focusing on elderly populations were rarely available. Other important risk factors, including prolonged mechanical ventilation, difficult weaning, and heart failure requiring ventilation, were also insufficiently examined. All subgroup analyses should be viewed as hypothesis-generating rather than definitive. Three studies with high risk of bias were included in the primary analysis, which may have influenced pooled estimates; however, we attempted to mitigate this through sensitivity analyses and restriction to higher-quality studies. Further, although multiple major databases were searched, the search strategy may not have captured all eligible RCTs, particularly those published outside the three databases searched or in languages other than English. Residual heterogeneity likely reflects interactions among risk factors, variability in HFNC and NIV protocols, and differences in standard ICU care across trials, all of which may have influenced reintubation rates and ICU length of stay.

Nonetheless, from a clinical perspective, the overall findings suggest that NIV may remain the preferred strategy in patients with the highest baseline risk of extubation failure, whereas HFNC may represent a reasonable alternative in selected lower-risk populations, pending confirmation from future trials.

## CONCLUSIONS

In the HIGH-FLOW OXY meta-analysis, no statistically significant differences were observed between HFNC and NIV in ICU mortality, length of stay, sepsis, nosocomial pneumonia, or 72-hour reintubation; however, estimates were imprecise and compatible with clinically important differences. When analyses were restricted to higher-quality RCTs, HFNC was associated with a higher risk of reintubation, suggesting the overall “no difference” finding may not be robust. Subgroup analyses, considered hypothesis-generating, suggested possible increased reintubation risk in very high-risk and nonoperative obese patients. Taken together, these findings suggest that the comparative effectiveness of HFNC and NIV may vary by patient phenotypes, and definitive conclusions cannot yet be drawn. Future phenotype-specific randomized trials are needed to better guide postextubation respiratory support strategies in high-risk critically ill populations.

## ACKNOWLEDGMENTS

We gratefully acknowledge the unwavering support and encouragement of our families and friends throughout our medical training, which has made this study possible. We also extend our sincere thanks to Dr. Rhanderson Cardoso of the Meta-analysis Academy for his expert guidance on meta-analytic methodology. Finally, we acknowledge the Faculty of Medicine at the University of Debrecen for providing foundational training in critical care medicine, which empowered us to undertake this research.

## Supplementary Material
