# Supplementary material for: High-Flow Nasal Cannula Versus Noninvasive Ventilation for Prevention of Reintubation in High-Risk Critically Ill Patients (HIGH-FLOW OXY): An Informative Systematic Review and Meta-Analysis
Source: Crit Care Explor. 2026 Aug 6;8(8):e1452. doi: 10.1097/CCE.0000000000001452 (PMC13446969; doi:10.1097/CCE.0000000000001452)
Supplement: Supplementary file 1 [file cc9-8-e1452-s001.pdf]

High Flow Nasal Canula versus Non-invasive Ventilation for Prevention of Reintubation in High-Risk Critically Ill patients (HIGH-FLOW OXY): An Informative Systematic Review and Meta-Analysis

Authors' full names:

Nhan Nguyen[1], MD; Nathalia Alves de Barros e Lyra*[2], MD; David Downes*[4], Bsc; Nghi Bao Tran*[1], MD; Vinh Quang Tri Ho*[1], MD; Yacin Zawam*[3], MD; Vy Ngoc Dan Nguyen[5], Bsc; Ha Duc Thien Le[1], Bsc; Jafar Aljazeeri*[6,7,8], MD, FCCP.

Authors' affiliation(s):

(1) University of Debrecen, Faculty of Medicine, Debrecen, Hungary.

(2) Faculdade Pernambucana de Saude, Recife, Pernambuco, Brazil.

(3) Southern Ohio Medical Center, Portsmouth, Ohio , USA.

(4) The University of New England, Department of Rural Medicine, Armidale, Australia.

(5) The University of Melbourne, Department of Nursing, Melbourne, Australia.

(6) University of Pittsburgh Medical Center, Harrisburg, USA.

(7) Drexel University College of Medicine, Philadelphia, USA.

(8) Medical City Hospital for Military and Security Services, Muscat, Oman.

* Drs Alves de Barros e Lyra, Downes, Tran, Ho, and Zawam contributed equally. Dr Aljazeeri is the senior author.

SUPPLEMENTARY FILE

List of contents:

1. **Supplementary Table 1. Baseline characteristics of included randomized controlled trials**
2. **Primary Pooled analysis of randomized controlled trials, Supplementary Figure 1 – Figure 4**
3. **Subgroup Analysis, Supplementary Figure 5 – Figure 10**
4. **Sensitivity analysis, all outcomes, Supplementary Figure 11 – Figure 16**
5. **Publication bias, assessment with Egger's test and funnel plots, Supplementary Figure 17 – Figure 26**
6. **Supplementary Table 2. Risk of Bias of included randomized controlled trials**
7. **Supplementary Table 3. Excluded studies**
8. **Supplementary Table 4. PRISMA checklist**
9. **Supplementary Table 5. GRADE table**

Study	*RENOVATE 2024*	*Tan 2020*	*Jing 2018*	*Cortegiani 2020*	*Tongyoo 2021*	*Theerawit 2021*	*Tseng 2023*	*Ketan 2024*	*Mokhtar 2025*	*Stéphan 2017*
	**HFNC / NIV**									
**Number of patients**	35 / 42	44 / 42	22 / 20	40 / 39	112 / 110	71 / 69	20 / 20	30 / 32	60 / 60	136 / 135
**Risk Factor of Extubation Failure**	AECOPD with hypercapnic respiratory failure	AECOPD with hypercapnic respiratory failure	AECOPD with hypercapnic respiratory failure	AECOPD with hypercapnic respiratory failure	Sepsis, with subgroups elderly and BMI above 30 kg/m^2	APACHE II score above 12 at extubation	Very High Risk, median of 5 risk factors	AECOPD with hypercapnic respiratory failure	General High Risk, < 4 risk factors	Obese, post-operative cardiothoracic surgery
**Age, years, mean**	74 / 71	68.4 / 71.4	77.4 / 73.9	74 / 77	62.6 / 63	68.2 / 71.2	74 / 73	65.3 / 65.3	59 / 60	63.4 / 64.5
**Male sex, %**	57.1 / 37.7	64.3 / 55.6	N/R	52.5 / 48.7	53.6 / 55.5	49.3 / 40.6	50 / 65	66.6 / 65.7	61 / 55	63.9 / 60.0
**BMI, kg/m^2, mean * or BMI above 25 kg/m^2, (%)**	N/R	N/R	N/R	30.5 / 26.7 *	23.6 / 24.1 *	20.7 / 21.5 *	N/R	N/R	30.5 / 29.8 *	34.4 / 34.2 *
**Severity score, mean APACHE II (1) / SAPS II (2) or 3 (3)**	63 / 58 [3] median	14 /13 [1] median	11.8 / 10.4 [1]	30 / 33[2]	15 / 15 [1] median	13 / 13.6 [1]	18 / 19 [1]	17.87 / 18.62 [1]	19.58/ 19.76 [1]	25.4 / 25.2 [2]
**Duration of intubation, mean, day**	N/R	98 / 114 median, hours	N/R	N/R	5 / 5 median	6.9 / 6.2	26 / 30	N/R	11.5 / 8 median	9.0 / 8.0 median
**Arterial pH, mean**	7.32 / 7.30	7.48 / 7.45	7.46 / 7.44	7.30 / 7.29	7.45 / 7.45	7.4 / 7.4	7.5 / 7.5	7.42 / 7.42	7.37 / 7.39 median	N/R
**$PaCO_2$, mmHg, mean**	55 / 64 median	50.5 / 55.0	53.2 / 53.7	73.7 / 72.0	34.2 / 35.3	35.1 / 35.8	39.2 / 38	48 / 47	43 / 42 median	N/R
**PaO_2/FiO_2 ratio, mean**	189 / 209 median	239.2 / 229.3	239.2 / 250.8	203.2 / 222.4	350.8 / 348.4	298.0 / 289.2	316 / 373	244.67 / 267.81	N/R	N/R
**COPD, %**	100	100	100	100	3 / 7	14.1 / 8	N/R	100	45 / 51.7	N/R
**Heart failure/heart disease, %**	25.7 / 19.0	N/R	N/R	N/R	6.3 / 4.5	29.6 / 37.7	N/R	N/R	58.3/ 65	N/R
**Diabetes mellitus, %**	34.3 / 31.0	22.7 / 33.3	N/R	N/R	43 (38.4) / 53 (48.2)	16 (43.2) / 21 (56.8)	N/R	N/R	40 (66.7) / 38 (63.3)	N/R
**CKD/renal failure, %**	5.7 / 4.8	27.3 / 19.0	N/R	N/R	30 (26.8) / 42 (38.2)	17 (23.9) / 18 (26.1)	N/R	N/R	21 (35) / 26 (43.3)	N/R
**Hypertension, %**	60.0 / 52.4	56.8 / 40.5	N/R	N/R	59.8 / 70.0	50 / 50	N/R	N/R	61.6 / 68.3	N/R

Supplementary Table 1. Baseline characteristics of included randomized clinical trials comparing high-flow nasal oxygen ($HFNO_2$) versus noninvasive ventilation (NIV) in critically ill patients at high risk for reintubation. $HFNO_2$/NIV columns represent the intervention and comparator groups, respectively. Severity score refers to APACHE II[1], SAPS II[2] or SAPS 3[3] and is reported at admission, extubation, or weaning according to each study protocol. Abbreviations: BMI, body mass index; CKD, chronic kidney disease; COPD, chronic obstructive pulmonary disease; $HFNO_2$, high-flow nasal

Study ***(CONTINUED)***	***Hernández 2016***	***Hernández 2022***	***Hernández 2025***	***Shang 2021***	***Kumari 2024***
**HFNC / NIV**					
**Number of patients**	290 / 314	90 / 92	72 / 72	24 / 24	30 / 30
**Risk Factor of Extubation Failure**	General high risk, below 4 risk factors, median of 3	Very high risk, 4 or above risk factors, median of 4 / 5	Obese, and general high risk, median of 3 risk factors	APACHE II score above 12 at extubation	More than 2 comorbidities, Charlson index at least 2
**Age, years, mean**	64.6 / 64.4	59.9 / 60.9	60 / 62	66.92 / 67.79	53 / 53.7
**Male sex, %**	64.1 / 64.3	55.6 / 72.8	52.5 / 48.7	20 / 23	70.0 / 66.7
**BMI, kg/m², mean * Or BMI above 25 kg/m², %**	BMI above 25 25.5 % / 23.6 %	BMI above 30 51.1 % / 53.3 %	BMI above 30, 100% BMI median of 33	22.32 / 22.87 median	27.16 / 28.09
**Severity score, mean APACHE II (1) / SAPS II (2) or 3 (3)**	63 / 62 [1]	19 /19 [1] median	15.9 / 16.9 [1]	Mean score between 12 and 24	N/R
**Duration of intubation, mean, day**	4 / 4 (days) median	5.5 / 4.5 (days) median	3 / 6	3.0 / 2.9	N/R
**Arterial pH, mean**	7.39 / 7.40	7.42 / 7.39	7.41 / 7.45	7.38 / 7.38	7.4 / 7.4
**$PaCO_2$, mmHg, mean**	41 / 39	41.6 / 44.2	40 / 39 median	39.75 / 39.65	41.7 / 42.5
**PaO_2/FiO_2 ratio, mean**	191 / 194	N/R	N/R	N/R	252.5 / 268.0
**COPD, %**	18.6 / 22.3	12.2 / 29.3	2.8 / 9.7		N/R
**Heart failure/heart disease, %**	32.4 / 32.5	24.4 / 41.3	16.7 / 19.4	20.83 / 16.67	N/R
**Diabetes mellitus, %**	30.7 / 28.7	N/R	29.2 / 19.4	N/R	N/R
**CKD/renal failure, %**	14.5 / 11.8	N/R	9.7 / 6.9	N/R	N/R
**Hypertension, %**	56.9 / 56.1	N/R	57.9 / 47.2	N/R	N/R

oxygen; NIV, noninvasive ventilation; $PaCO_2$, arterial partial pressure of carbon dioxide; PaO_2/FiO_2, ratio of arterial oxygen partial pressure to fraction of inspired oxygen; NR indicates not reported. * mean value.

Supplementary Table 1. Baseline characteristics of included randomized clinical trials comparing high-flow nasal oxygen ($HFNO_2$) versus noninvasive ventilation (NIV) in critically ill patients at high risk for reintubation. $HFNO_2$/NIV columns represent the intervention and comparator groups, respectively. Severity score refers to APACHE II[1], SAPS II[2] or SAPS 3[3] and is reported at admission, extubation, or weaning according to each study protocol. Abbreviations: BMI, body mass index; CKD, chronic kidney disease; COPD, chronic obstructive pulmonary disease; $HFNO_2$, high-flow nasal oxygen; NIV, noninvasive ventilation; $PaCO_2$, arterial partial pressure of carbon dioxide; PaO_2/FiO_2, ratio of arterial oxygen partial pressure to fraction of inspired oxygen; NR indicates not reported.

Pooled Primary Meta-Analysis (Randomized Controlled Trials)

Figure 2. Short-termed Reintubation, within 72 hours.

	HFNC		NIV			Odds ratio	Odds ratio
**Study or Subgroup**	**Events**	**Total**	**Events**	**Total**	**Weight**	**IV, Random, 95% CI**	**IV, Random, 95% CI**
Cortegiani et al 2020	1	40	1	39	1.1%	0.97 [0.06 , 16.14]	
Hernandez et al 2016	66	290	60	314	26.9%	1.25 [0.84 , 1.85]	
Hernandez et al 2022	26	90	18	92	13.6%	1.67 [0.84 , 3.32]	
Hernandez et al 2025	20	72	12	72	10.7%	1.92 [0.86 , 4.31]	
Jing et al 2019	2	22	1	20	1.4%	1.90 [0.16 , 22.72]	
Ketan et al 2024	2	30	8	32	3.1%	0.21 [0.04 , 1.11]	
Kumari et al 2024	14	30	6	30	5.9%	3.50 [1.11 , 11.02]	
Mokhtar et al 2025	13	60	15	60	9.9%	0.83 [0.36 , 1.94]	
Shang et al 2021	0	24	1	24	0.8%	0.32 [0.01 , 8.25]	
Stephan et al 2017	5	135	8	136	5.9%	0.62 [0.20 , 1.93]	
Tan et al 2020	6	44	6	42	5.3%	0.95 [0.28 , 3.21]	
Theerawit et al 2021	5	71	6	69	5.2%	0.80 [0.23 , 2.74]	
Tongyoo et al 2021	12	60	13	58	9.2%	0.87 [0.36 , 2.09]	
Tseng et al 2023	3	20	0	20	0.9%	8.20 [0.40 , 169.90]	
**Total (Wald[a])**		**988**		**1008**	**100.0%**	**1.19 [0.88 , 1.59]**	
Total events:	175		155				

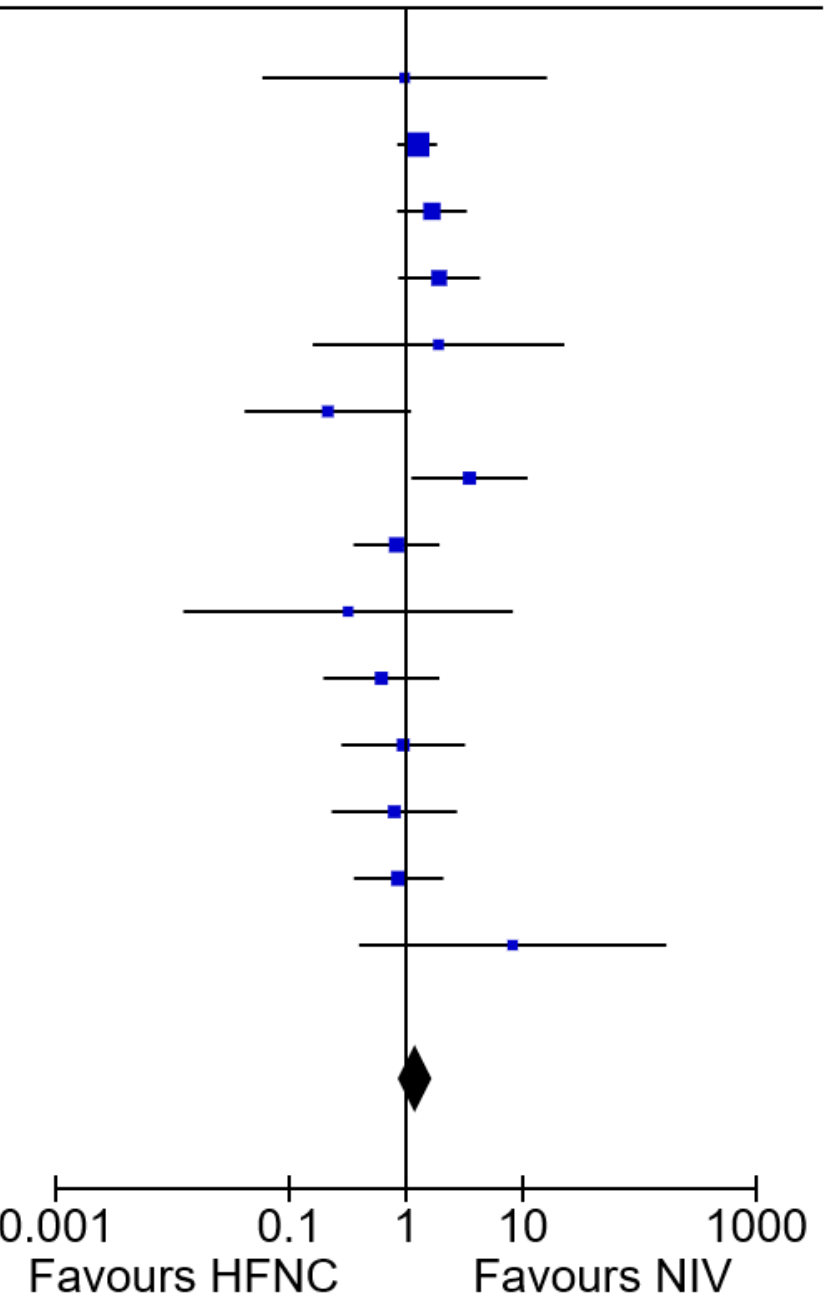

Test for overall effect: Z = 1.12 (P = 0.26)

Heterogeneity: Tau² (DL[b]) = 0.04; Chi² = 15.27, df = 13 (P = 0.29); I² = 15%

Footnotes

[a]CI calculated by Wald-type method.

[b]Tau² calculated by DerSimonian and Laird method.

Supplementary Figure 1. ICU Mortality

Study or Subgroup	HFNC Events	HFNC Total	NIV Events	NIV Total	Weight	Odds ratio IV, Random, 95% CI
Hernandez et al 2022	4	90	12	92	22.0%	0.31 [0.10 , 1.00]
Hernandez et al 2025	8	72	10	72	28.1%	0.78 [0.29 , 2.09]
Hernandez et al post hoc analysis 2016	19	290	18	314	46.3%	1.15 [0.59 , 2.24]
Shang et al 2021	0	20	1	20	3.5%	0.32 [0.01 , 8.26]
Tseng et al 2023	0	24	0	24		Not estimable
**Total (Wald[a])**		**496**		**522**	**100.0%**	**0.74 [0.40 , 1.38]**
Total events:	31		41			

Test for overall effect: Z = 0.95 (P = 0.34)

Heterogeneity: Tau² (DL[b]) = 0.10; Chi² = 3.99, df = 3 (P = 0.26); I² = 25%

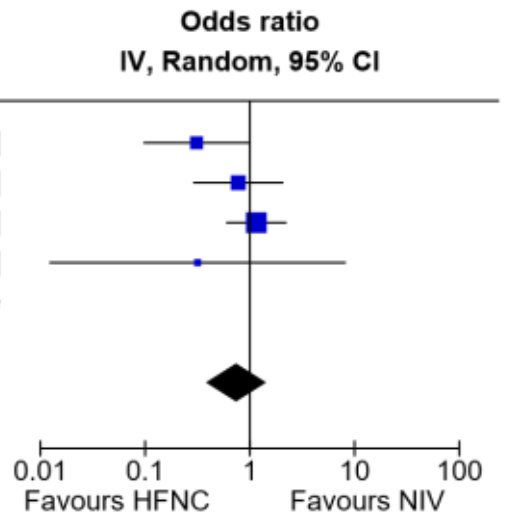

Footnotes

[a]CI calculated by Wald-type method.

[b]Tau² calculated by DerSimonian and Laird method.

Supplementary Figure 2. Sepsis

Study or Subgroup	HFNC Events	HFNC Total	NIV Events	NIV Total	Weight	Odds ratio IV, Random, 95% CI
Hernandez et al 2016	6	290	4	314	22.2%	1.64 [0.46 , 5.86]
Hernandez et al 2022	3	90	4	92	15.5%	0.76 [0.16 , 3.49]
Hernandez et al 2025	3	72	2	72	10.9%	1.52 [0.25 , 9.39]
Mokhtar et al 2025	16	60	13	60	51.3%	1.31 [0.57 , 3.04]
**Total (Wald[a])**		**512**		**538**	**100.0%**	**1.29 [0.71 , 2.35]**
Total events:	28		23			

Test for overall effect: Z = 0.82 (P = 0.41)

Heterogeneity: Tau² (DL[b]) = 0.00; Chi² = 0.63, df = 3 (P = 0.89); I² = 0%

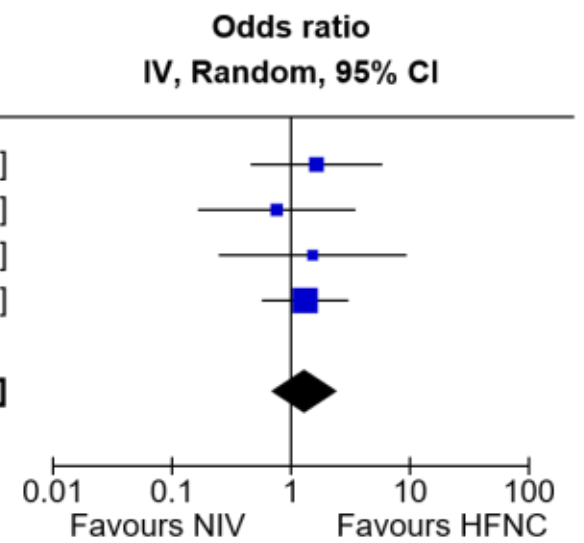

Footnotes

[a]CI calculated by Wald-type method.

[b]Tau² calculated by DerSimonian and Laird method.

Supplementary Figure 3. Nosocomial Pneumonia

Study or Subgroup	HFNC Events	HFNC Total	NIV Events	NIV Total	Weight	Odds ratio IV, Random, 95% CI
Hernandez et al 2016	12	290	17	314	29.3%	0.75 [0.35 , 1.61]
Hernandez et al 2022	7	90	4	92	10.5%	1.86 [0.52 , 6.57]
Hernandez et al 2025	5	72	5	72	10.2%	1.00 [0.28 , 3.61]
Mokhtar et al 2025	10	60	9	60	17.5%	1.13 [0.42 , 3.02]
Stephan et al 2017	17	135	17	136	32.5%	1.01 [0.49 , 2.07]
**Total (Wald[a])**		**647**		**674**	**100.0%**	**1.01 [0.67 , 1.52]**
Total events:	51		52			

Test for overall effect: Z = 0.03 (P = 0.97)

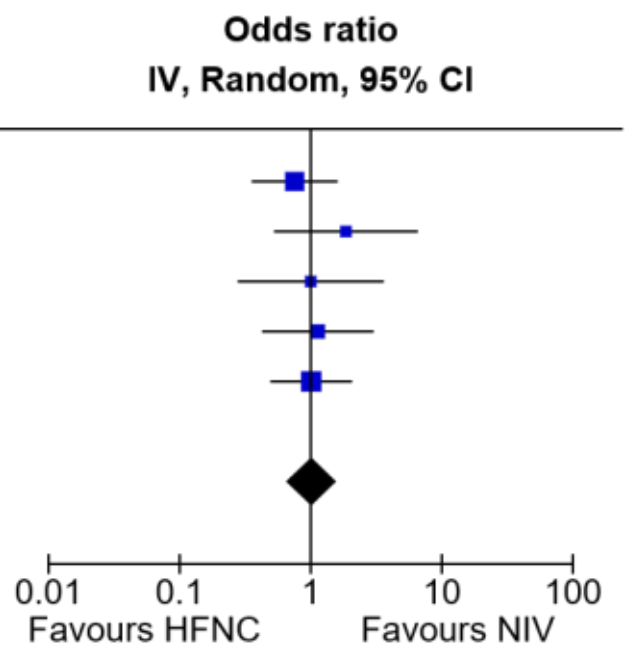

Heterogeneity: Tau² (DL[b]) = 0.00; Chi² = 1.51, df = 4 (P = 0.82); I² = 0%

Footnotes

[a]CI calculated by Wald-type method.

[b]Tau² calculated by DerSimonian and Laird method.

Supplementary Figure 4. ICU length of stay

	HFNC			NIV				Mean difference
**Study or Subgroup**	**Mean**	**SD**	**Total**	**Mean**	**SD**	**Total**	**Weight**	**IV, Random, 95% CI**
Hernandez et al 2016	3.75	1.4506	290	4.75	2.0259	314	15.8%	-1.00 [-1.28 , -0.72]
Hernandez et al 2022	12.675	3.5566	90	9.5	3.182	92	13.5%	3.18 [2.19 , 4.16]
Hernandez et al 2025	8.25	4.0543	72	12	16.4583	72	4.0%	-3.75 [-7.67 , 0.17]
Jing et al 2019	8.5	3.5	22	9.4	4.8	20	7.0%	-0.90 [-3.46 , 1.66]
Mokhtar et al 2025	16	1.744	60	17.5	2.2913	60	14.6%	-1.50 [-2.23 , -0.77]
RENOVATE 2025	3.25	1.5478	32	4.25	1.5207	39	14.6%	-1.00 [-1.72 , -0.28]
Shang et al 2021	6.88375	1.711667	24	7.19	1.585416	24	13.7%	-0.31 [-1.24 , 0.63]
Tan et al 2020	7.75	1.1815	44	8.825	1.8358	42	14.8%	-1.07 [-1.73 , -0.42]
Tseng et al 2023	18	10	20	17	8.9	20	2.0%	1.00 [-4.87 , 6.87]
**Total (Wald[a])**			**654**			**683**	**100.0%**	**-0.49 [-1.38 , 0.41]**

Mean difference
IV, Random, 95% CI

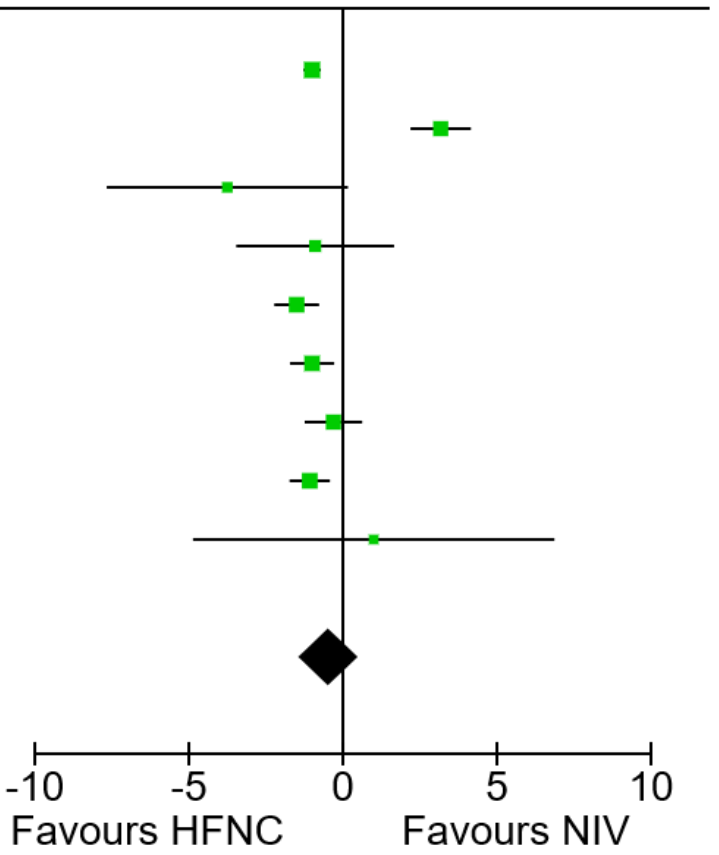

Test for overall effect: Z = 1.06 (P = 0.29)

Heterogeneity: Tau^2 (DL[b]) = 1.30; Chi^2 = 73.14, df = 8 (P < 0.00001); I^2 = 89%

Footnotes

[a]CI calculated by Wald-type method.

[b]Tau^2 calculated by DerSimonian and Laird method.

<u>**Subgroup analysis– Reintubation outcome within first 3 days after extubation**</u>

A. Very high risk (risk factors of 4 and above)

Supplementary Figure 5.Very High Risk

	HFNC		NIV			Odds ratio
**Study or Subgroup**	**Events**	**Total**	**Events**	**Total**	**Weight**	**IV, Random, 95% CI**
Hernandez et al 2022	26	90	18	92	41.1%	1.67 [0.84 , 3.32]
Hernandez et al post hoc analysis 2016	43	114	27	94	56.7%	1.50 [0.84 , 2.70]
Tseng et al 2023	3	20	0	20	2.1%	8.20 [0.40 , 169.90]
**Total (Wald[a])**		**224**		**206**	**100.0%**	**1.63 [1.05 , 2.53]**
Total events:	72		45			
Test for overall effect: Z = 2.16 (P = 0.03)						

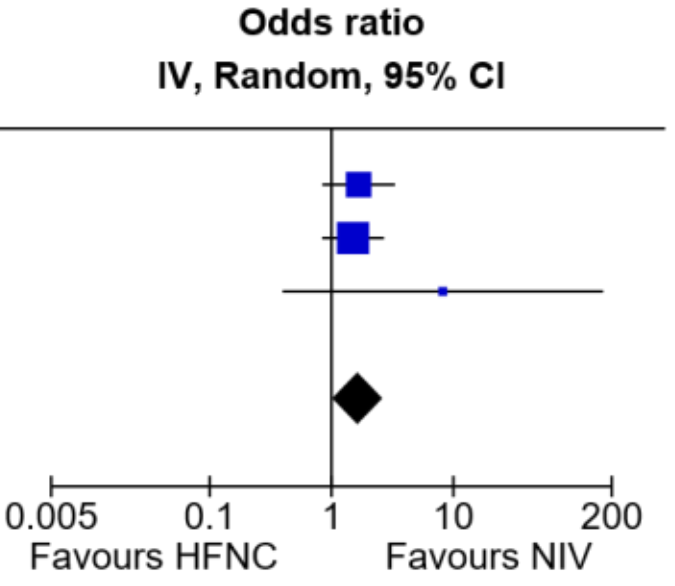

Heterogeneity: Tau² (DL[b]) = 0.00; Chi² = 1.17, df = 2 (P = 0.56); I² = 0%

Footnotes

[a]CI calculated by Wald-type method.

[b]Tau² calculated by DerSimonian and Laird method.

B. Obesity

With Kumari et al. 2024

Supplementary Figure 6. Obese, INCLUDE Post-operative critically ill patients

Study or Subgroup	HFNC Events	HFNC Total	NIV Events	NIV Total	Weight	Odds ratio IV, Random, 95% CI
Hernandez et al 2025	20	72	12	72	27.7%	1.92 [0.86 , 4.31]
Hernandez et al post hoc analysis 2016	25	74	11	74	27.8%	2.92 [1.31 , 6.51]
Kumari et al 2024	14	30	6	30	19.0%	3.50 [1.11 , 11.02]
Stephan et al 2017	5	135	8	136	19.1%	0.62 [0.20 , 1.93]
Tongyoo et al 2021	4	12	1	15	6.4%	7.00 [0.66 , 73.93]
**Total (Wald[a])**		**323**		**327**	**100.0%**	**2.11 [1.12 , 4.00]**
Total events:	68		38			
Test for overall effect: Z = 2.31 (P = 0.02)						

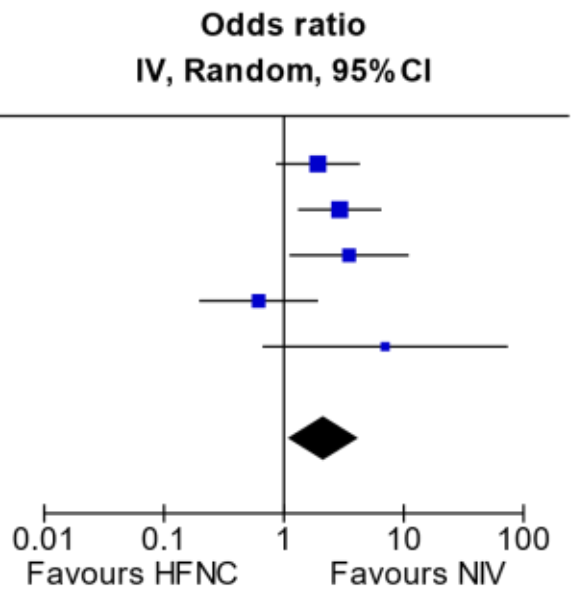

Heterogeneity: Tau^2 (DL[b]) = 0.21; Chi^2 = 6.89, df = 4 (P = 0.14); I^2 = 42%

Footnotes

[a]CI calculated by Wald-type method.

[b]Tau^2 calculated by DerSimonian and Laird method.

Supplementary Figure 7. Obese, EXCLUDE Post-operative critically ill patients

Study or Subgroup	HFNC Events	HFNC Total	NIV Events	NIV Total	Weight	Odds ratio IV, Random, 95% CI
✓ Hernandez et al 2025	20	72	12	72	38.1%	1.92 [0.86 , 4.31]
✓ Hernandez et al post hoc analysis 2016	25	74	11	74	38.6%	2.92 [1.31 , 6.51]
✓ Kumari et al 2024	14	30	6	30	18.8%	3.50 [1.11 , 11.02]
✗ Stephan et al 2017	5	135	8	136	0.0%	0.62 [0.20 , 1.93]
✓ Tongyoo et al 2021	4	12	1	15	4.5%	7.00 [0.66 , 73.93]
**Total (Wald[a])**		**188**		**191**	**100.0%**	**2.68 [1.63 , 4.41]**
Total events:	63		30			

Test for overall effect: Z = 3.88 (P = 0.0001)

Heterogeneity: Tau² (DL[b]) = 0.00; Chi² = 1.54, df = 3 (P = 0.67); I² = 0%

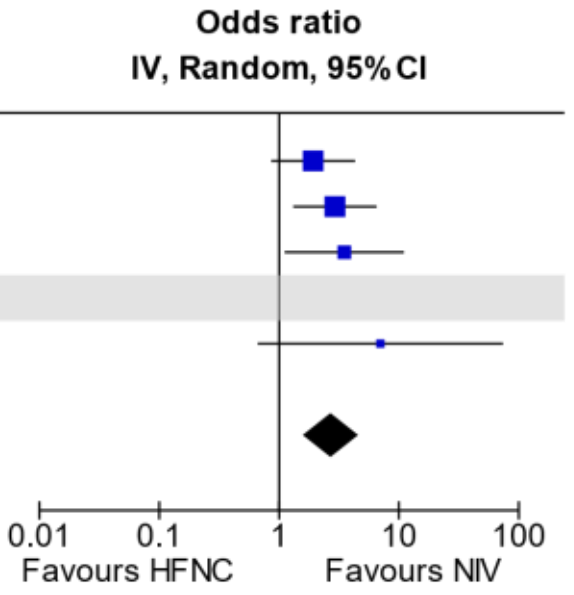

Footnotes
[a]CI calculated by Wald-type method.
[b]Tau² calculated by DerSimonian and Laird method.

Without Kumari et al. 2024

Supplementary Figure 8. Obese, INCLUDE Post-operative critically ill patients

Study or Subgroup	HFNC Events	HFNC Total	NIV Events	NIV Total	Weight	Odds ratio IV, Random, 95% CI
Hernandez et al 2025	20	72	12	72	33.4%	1.92 [0.86 , 4.31]
Hernandez et al post hoc analysis 2016	25	74	11	74	33.5%	2.92 [1.31 , 6.51]
Stephan et al 2017	5	135	8	136	24.3%	0.62 [0.20 , 1.93]
Tongyoo et al 2021	4	12	1	15	8.8%	7.00 [0.66 , 73.93]
**Total (Wald[a])**		**293**		**297**	**100.0%**	**1.88 [0.87 , 4.05]**
Total events:	54		32			

Test for overall effect: Z = 1.61 (P = 0.11)

Heterogeneity: Tau² (DL[b]) = 0.29; Chi² = 6.01, df = 3 (P = 0.11); I² = 50%

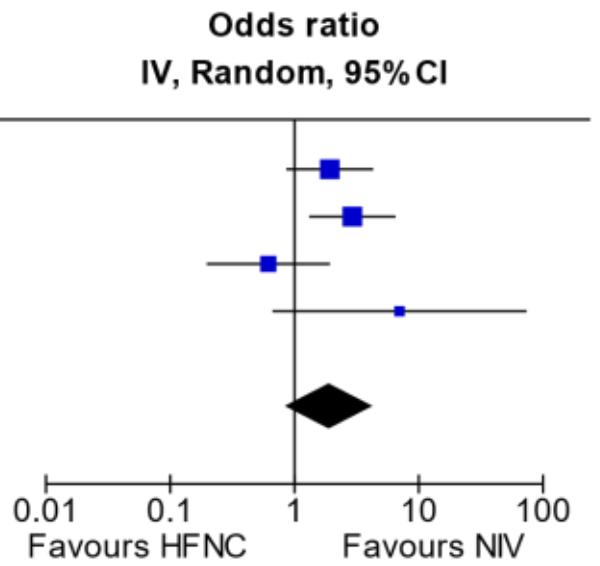

Footnotes
[a]CI calculated by Wald-type method.
[b]Tau² calculated by DerSimonian and Laird method.

Supplementary Figure 9. Obese, EXCLUDE Post-operative critically ill patients

Study or Subgroup	HFNC Events	HFNC Total	NIV Events	NIV Total	Weight	Odds ratio IV, Random, 95% CI
Hernandez et al 2025	20	72	12	72	47.0%	1.92 [0.86 , 4.31]
Hernandez et al post hoc analysis 2016	25	74	11	74	47.5%	2.92 [1.31 , 6.51]
Stephan et al 2017	5	135	8	136	0.0%	0.62 [0.20 , 1.93]
Tongyoo et al 2021	4	12	1	15	5.5%	7.00 [0.66 , 73.93]
**Total (Wald[a])**		**158**		**161**	**100.0%**	**2.52 [1.45 , 4.38]**
Total events:	49		24			

Test for overall effect: Z = 3.28 (P = 0.001)

Heterogeneity: Tau^2 (DL[b]) = 0.00; Chi^2 = 1.28, df = 2 (P = 0.53); I^2 = 0%

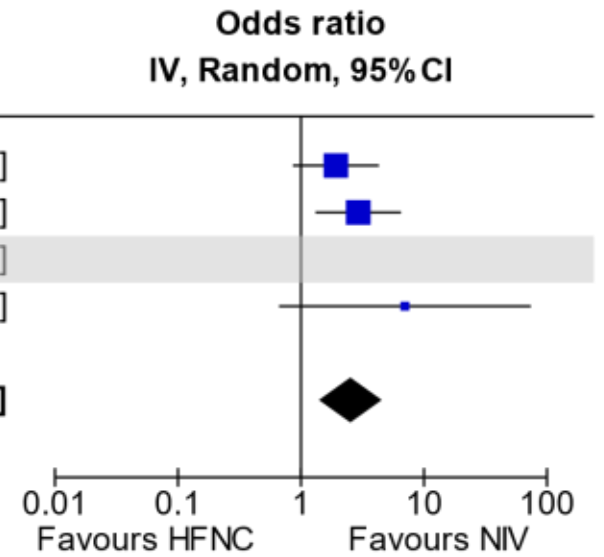

Footnotes

[a]CI calculated by Wald-type method.

[b]Tau² calculated by DerSimonian and Laird method.

C. AECOPD with hypercapnic respiratory failure

Supplementary Figure 10. Acute Exarcebation of COPD with hypercapnic respiratory failure

	HFNC		NIV			Odds ratio	Odds ratio
**Study or Subgroup**	**Events**	**Total**	**Events**	**Total**	**Weight**	**IV, Random, 95% CI**	**IV, Random, 95% CI**
Cortegiani et al 2020	1	39	1	40	9.5%	1.03 [0.06 , 17.01]	
Jing et al 2019	2	22	1	20	12.2%	1.90 [0.16 , 22.72]	
Ketan et al 2024	2	30	8	32	27.8%	0.21 [0.04 , 1.11]	
Tan et al 2020	6	44	6	42	50.5%	0.95 [0.28 , 3.21]	
**Total (Wald[a])**		**135**		**134**	**100.0%**	**0.69 [0.29 , 1.63]**	
Total events:	11		16				

Test for overall effect: Z = 0.85 (P = 0.40)

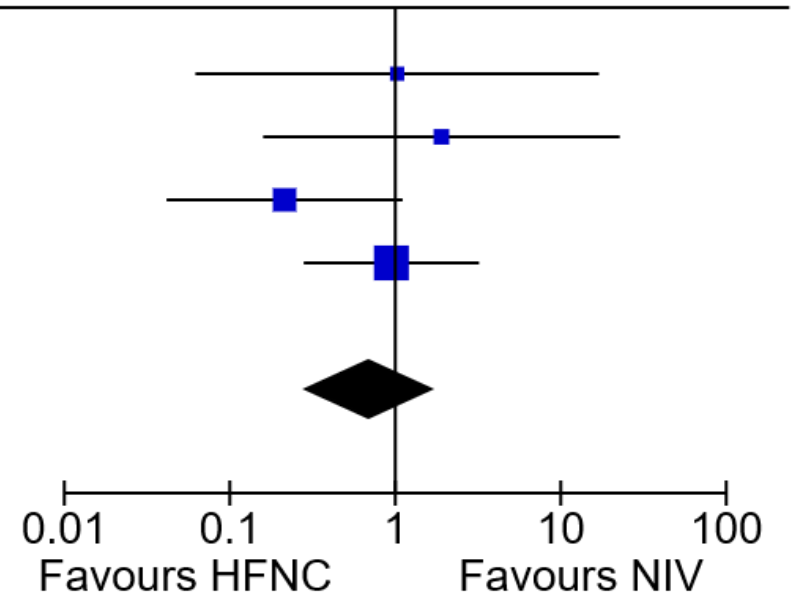

Heterogeneity: Tau² (DL[b]) = 0.00; Chi² = 2.92, df = 3 (P = 0.40); I² = 0%

Footnotes

[a]CI calculated by Wald-type method.

[b]Tau² calculated by DerSimonian and Laird method.

Sensitivity analysis of all outcomes

Exclusion of High Risk of Bias Studies, Ketan et al. 2024, Shang et al. 2021 and Mokhtar et al. 2025

Supplementary Figure 11. Short-termed Reintubation, within 72 hours.

	HFNC		NIV			Odds ratio
**Study or Subgroup**	**Events**	**Total**	**Events**	**Total**	**Weight**	**IV, Random, 95% CI**
✓ Cortegiani et al 2020	1	40	1	39	0.9%	0.97 [0.06 , 16.14]
✓ Hernandez et al 2016	66	290	60	314	44.2%	1.25 [0.84 , 1.85]
✓ Hernandez et al 2022	26	90	18	92	14.4%	1.67 [0.84 , 3.32]
✓ Hernandez et al 2025	20	72	12	72	10.5%	1.92 [0.86 , 4.31]
✓ Jing et al 2019	2	22	1	20	1.1%	1.90 [0.16 , 22.72]
✕ Ketan et al 2024	2	30	8	32	0.0%	0.21 [0.04 , 1.11]
✓ Kumari et al 2024	14	30	6	30	5.2%	3.50 [1.11 , 11.02]
✕ Mokhtar et al 2025	13	60	15	60	0.0%	0.83 [0.36 , 1.94]
✕ Shang et al 2021	0	24	1	24	0.0%	0.32 [0.01 , 8.25]
✓ Stephan et al 2017	5	135	8	136	5.2%	0.62 [0.20 , 1.93]
✓ Tan et al 2020	6	44	6	42	4.6%	0.95 [0.28 , 3.21]
✓ Theerawit et al 2021	5	71	6	69	4.5%	0.80 [0.23 , 2.74]
✓ Tongyoo et al 2021	12	60	13	58	8.7%	0.87 [0.36 , 2.09]
✓ Tseng et al 2023	3	20	0	20	0.7%	8.20 [0.40 , 169.90]
**Total (Wald[a])**		**874**		**892**	**100.0%**	**1.32 [1.02 , 1.71]**
Total events:	160		131			

Test for overall effect: Z = 2.08 (P = 0.04)

Heterogeneity: Tau² (DL[b]) = 0.00; Chi² = 9.18, df = 10 (P = 0.51); I² = 0%

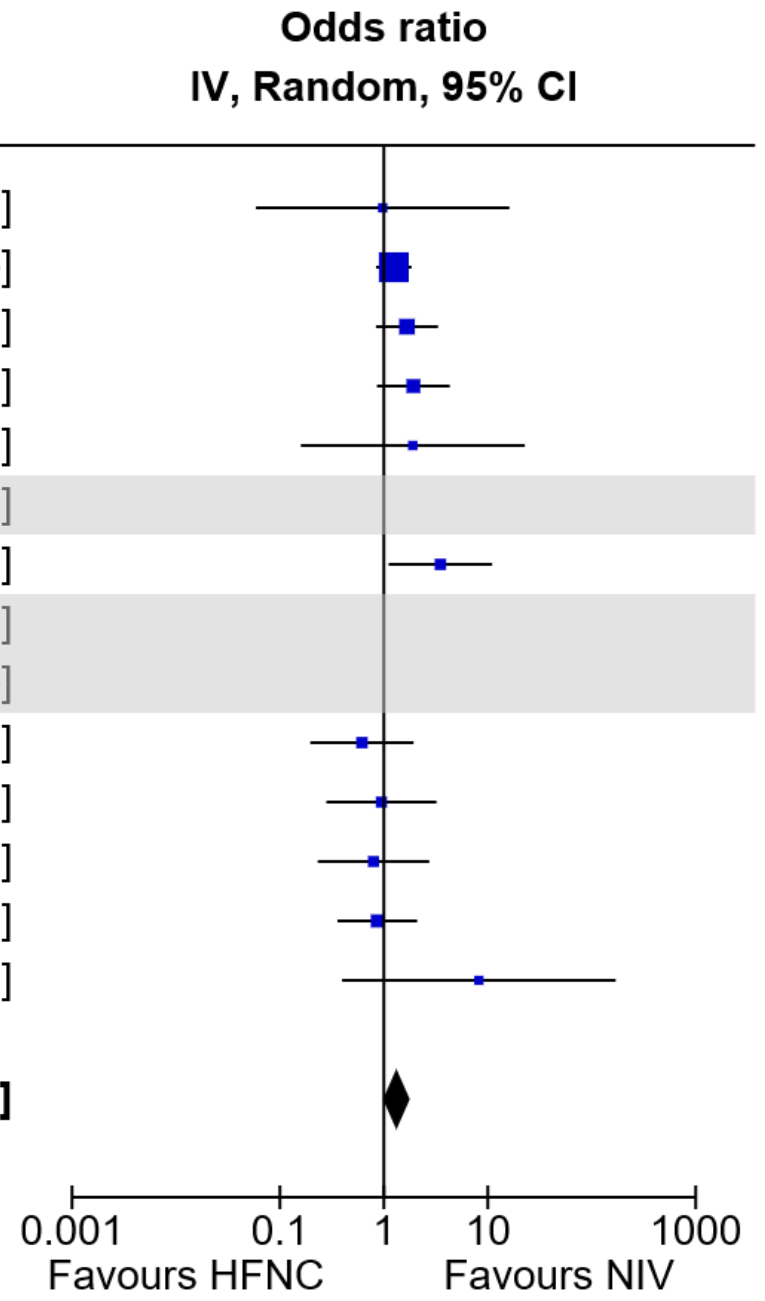

Footnotes

[a]CI calculated by Wald-type method.

[b]Tau² calculated by DerSimonian and Laird method.

Supplementary Figure 12. Short-termed Reintubation within 72 hours, without very high risk group

Study or Subgroup	HFNC Events	HFNC Total	NIV Events	NIV Total	Weight	Odds ratio IV, Random, 95% CI
✓ Cortegiani et al 2020	1	40	1	39	2.0%	0.97 [0.06 , 16.14]
✕ Hernandez et al 2022	26	90	18	92	0.0%	1.67 [0.84 , 3.32]
✓ Hernandez et al 2025	20	72	12	72	17.0%	1.92 [0.86 , 4.31]
✓ Hernandez et al post hoc analysis 2016	24	200	33	196	26.0%	0.67 [0.38 , 1.19]
✓ Jing et al 2019	2	22	1	20	2.5%	1.90 [0.16 , 22.72]
✕ Ketan et al 2024	2	30	8	32	0.0%	0.21 [0.04 , 1.11]
✓ Kumari et al 2024	14	30	6	30	10.0%	3.50 [1.11 , 11.02]
✕ Mokhtar et al 2025	13	60	15	60	0.0%	0.83 [0.36 , 1.94]
✕ Shang et al 2021	0	24	1	24	0.0%	0.32 [0.01 , 8.25]
✓ Stephan et al 2017	5	135	8	136	10.0%	0.62 [0.20 , 1.93]
✓ Tan et al 2020	6	44	6	42	9.0%	0.95 [0.28 , 3.21]
✓ Theerawit et al 2021	5	71	6	69	8.8%	0.80 [0.23 , 2.74]
✓ Tongyoo et al 2021	12	60	13	58	14.9%	0.87 [0.36 , 2.09]
✕ Tseng et al 2023	3	20	0	20	0.0%	8.20 [0.40 , 169.90]
**Total (Wald[a])**		**674**		**662**	**100.0%**	**1.05 [0.71 , 1.57]**
Total events:	89		86			
Test for overall effect: Z = 0.26 (P = 0.79)						

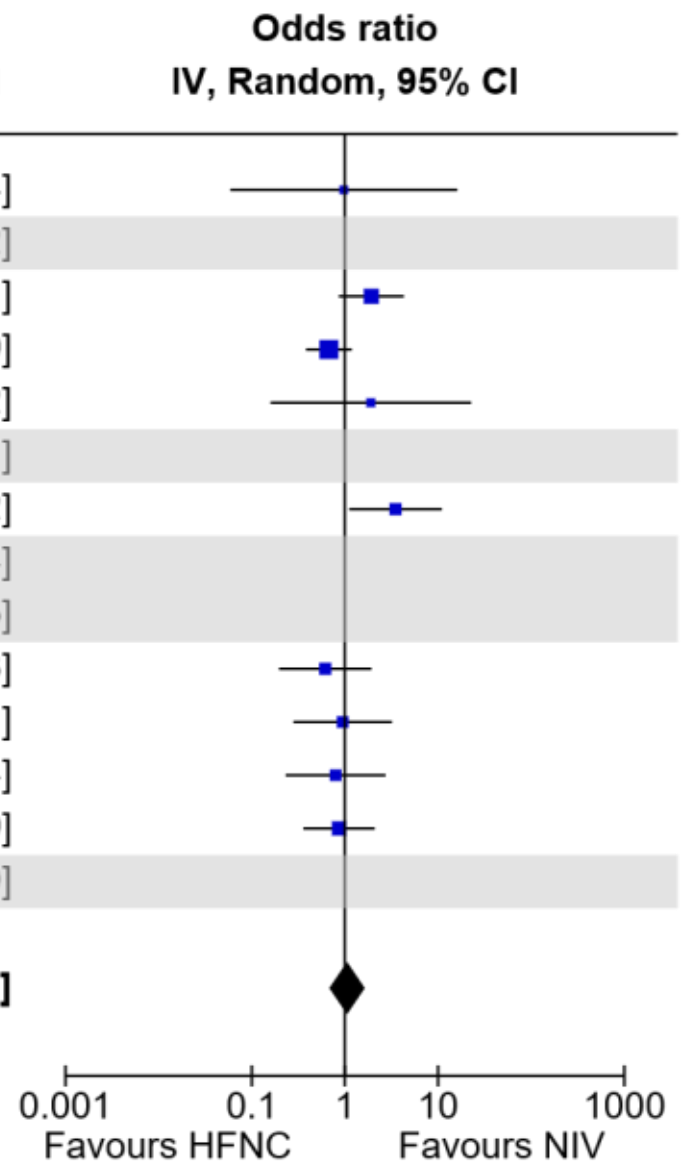

Heterogeneity: Tau² (DL[b]) = 0.08; Chi² = 10.16, df = 8 (P = 0.25); I² = 21%

Footnotes

[a]CI calculated by Wald-type method.

[b]Tau² calculated by DerSimonian and Laird method.

Supplementary Figure 13. Sepsis

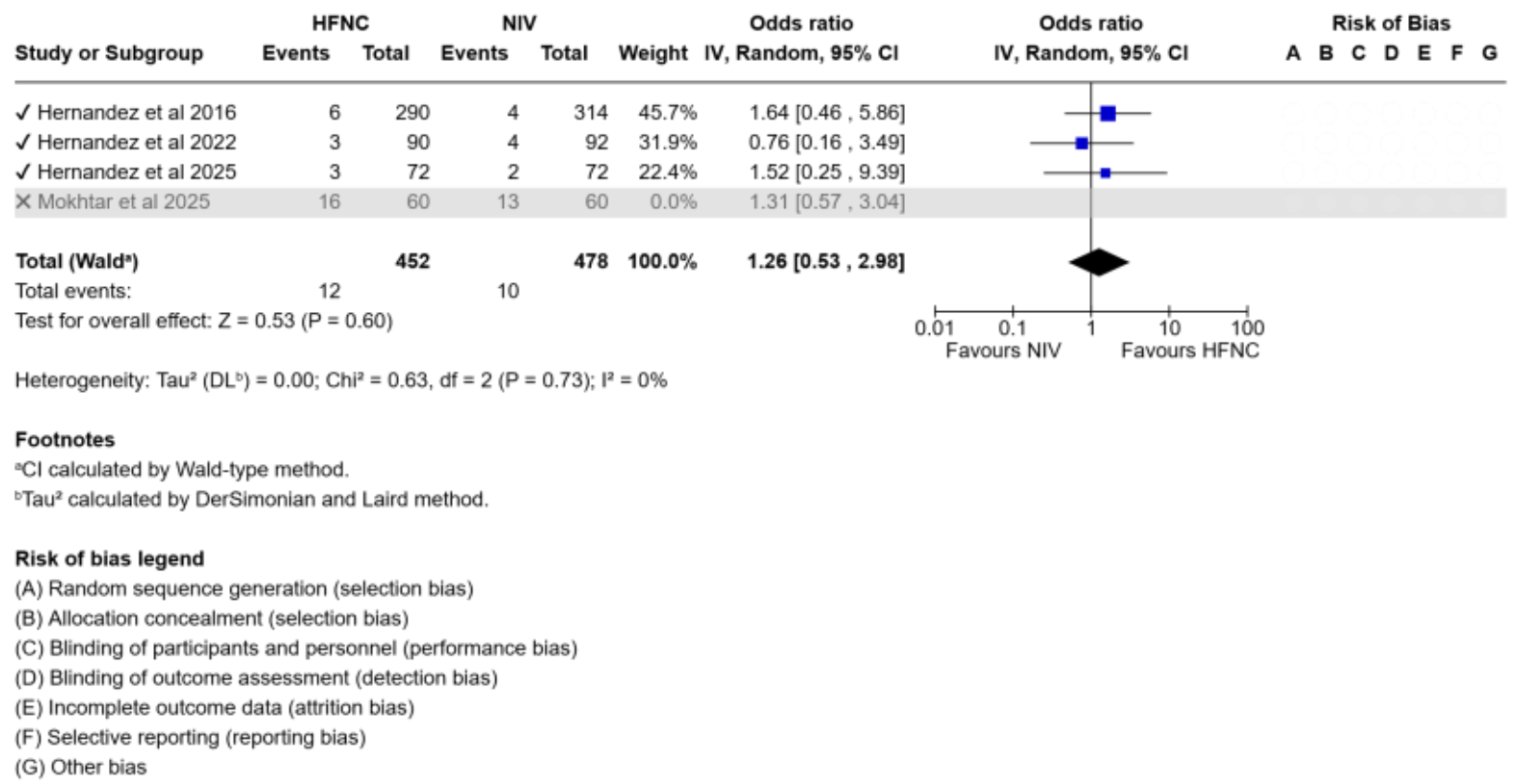

Supplementary Figure 14. Nosocomial Pneumonia

Study or Subgroup	HFNC Events	HFNC Total	NIV Events	NIV Total	Weight	Odds ratio IV, Random, 95% CI	Odds ratio IV, Random, 95% CI	Risk of Bias A	B	C	D	E	F	G
✓ Hernandez et al 2016	12	290	17	314	35.5%	0.75 [0.35 , 1.61]								
✓ Hernandez et al 2022	7	90	4	92	12.7%	1.86 [0.52 , 6.57]								
✓ Hernandez et al 2025	5	72	5	72	12.3%	1.00 [0.28 , 3.61]								
✕ Mokhtar et al 2025	10	60	9	60	0.0%	1.13 [0.42 , 3.02]								
✓ Stephan et al 2017	17	135	17	136	39.4%	1.01 [0.49 , 2.07]								
**Total (Wald[a])**		**587**		**614**	**100.0%**	**0.98 [0.63 , 1.54]**								
Total events:	41		43											

Test for overall effect: Z = 0.08 (P = 0.94)

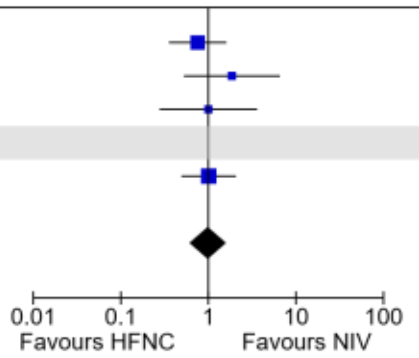

Heterogeneity: Tau^2 (DL[b]) = 0.00; Chi^2 = 1.45, df = 3 (P = 0.69); I^2 = 0%

Footnotes

[a]CI calculated by Wald-type method.

[b]Tau^2 calculated by DerSimonian and Laird method.

Risk of bias legend

(A) Random sequence generation (selection bias)
(B) Allocation concealment (selection bias)
(C) Blinding of participants and personnel (performance bias)
(D) Blinding of outcome assessment (detection bias)
(E) Incomplete outcome data (attrition bias)
(F) Selective reporting (reporting bias)
(G) Other bias

Supplementary Figure 15. ICU length of stay, with Mokhtar et al. 2025

Study or Subgroup	HFNC Mean	HFNC SD	HFNC Total	NIV Mean	NIV SD	NIV Total	Weight	Mean difference IV, Random, 95% CI
**1.4.1 General high risk subgroup, below 4 risk factors**								
Hernandez et al 2016	3.75	1.4506	290	4.75	2.0259	314	15.8%	-1.00 [-1.28 , -0.72]
Mokhtar et al 2025	16	1.744	60	17.5	2.2913	60	14.6%	-1.50 [-2.23 , -0.77]
**Subtotal (Wald[a])**			**350**			**374**	**30.4%**	**-1.13 [-1.56 , -0.70]**
Test for overall effect: Z = 5.14 (P < 0.00001)								
Heterogeneity: Tau² (DL[b]) = 0.05; Chi² = 1.58, df = 1 (P = 0.21); I² = 37%								
**1.4.2 Acute exarcebations of COPD with hypercapnic respiratory failure**								
Jing et al 2019	8.5	3.5	22	9.4	4.8	20	7.0%	-0.90 [-3.46 , 1.66]
RENOVATE 2025	3.25	1.5478	32	4.25	1.5207	39	14.6%	-1.00 [-1.72 , -0.28]
Tan et al 2020	7.75	1.1815	44	8.825	1.8358	42	14.8%	-1.07 [-1.73 , -0.42]
**Subtotal (Wald[a])**			**98**			**101**	**36.4%**	**-1.04 [-1.51 , -0.56]**
Test for overall effect: Z = 4.27 (P < 0.0001)								
Heterogeneity: Tau² (DL[b]) = 0.00; Chi² = 0.03, df = 2 (P = 0.98); I² = 0%								
**1.4.3 Very high risk subgroup, 4 and above risk factors**								
Hernandez et al 2022	12.675	3.5566	90	9.5	3.182	92	13.5%	3.18 [2.19 , 4.16]
Tseng et al 2023	18	10	20	17	8.9	20	2.0%	1.00 [-4.87 , 6.87]
**Subtotal (Wald[a])**			**110**			**112**	**15.5%**	**3.12 [2.15 , 4.08]**
Test for overall effect: Z = 6.31 (P < 0.00001)								
Heterogeneity: Tau² (DL[b]) = 0.00; Chi² = 0.51, df = 1 (P = 0.47); I² = 0%								
**1.4.4 Non-operative obese subgroup**								
Hernandez et al 2025	8.25	4.0543	72	12	16.4583	72	4.0%	-3.75 [-7.67 , 0.17]
**Subtotal**			**72**			**72**	**4.0%**	**-3.75 [-7.67 , 0.17]**
Test for overall effect: Z = 1.88 (P = 0.06)								
Heterogeneity: Not applicable								
**1.4.5 APACHE II score above 12 at extubation**								
Shang et al 2021	6.88375	1.711667	24	7.19	1.585416	24	13.7%	-0.31 [-1.24 , 0.63]
**Subtotal**			**24**			**24**	**13.7%**	**-0.31 [-1.24 , 0.63]**
Test for overall effect: Z = 0.64 (P = 0.52)								
Heterogeneity: Not applicable								
**Total (Wald[a])**			**654**			**683**	**100.0%**	**-0.49 [-1.38 , 0.41]**

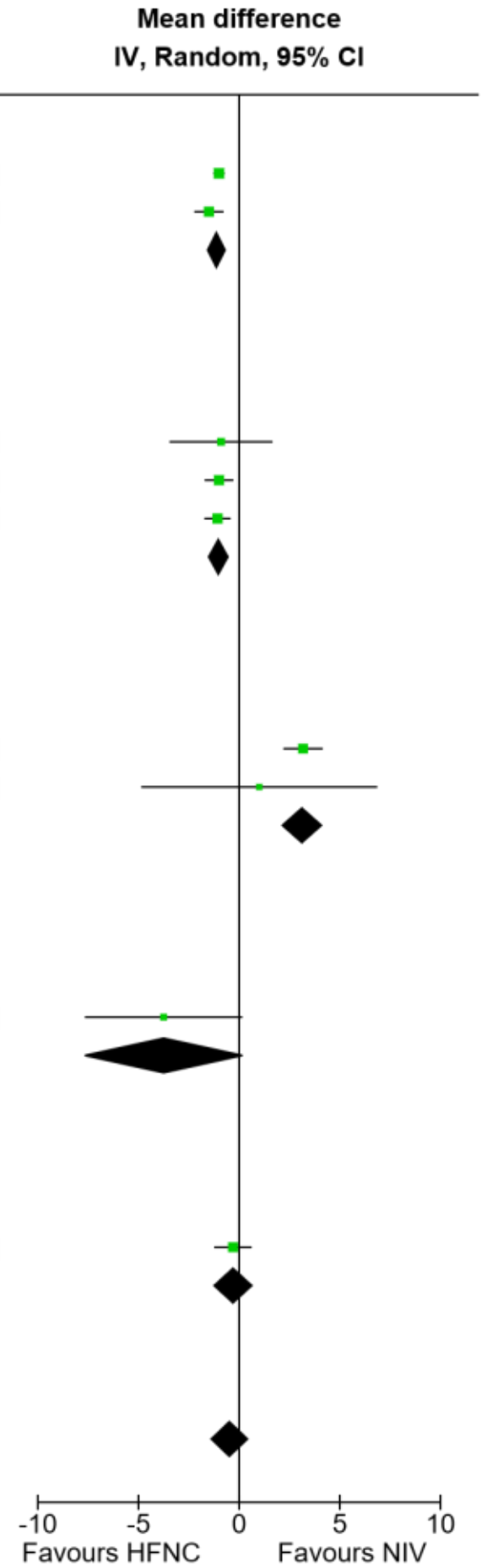

Test for overall effect: Z = 1.06 (P = 0.29)
Test for subgroup differences: Chi² = 68.41, df = 4 (P < 0.00001), I² = 94.2%
Heterogeneity: Tau² (DL[b]) = 1.30; Chi² = 73.14, df = 8 (P < 0.00001); I² = 89%

Footnotes
[a]CI calculated by Wald-type method.
[b]Tau² calculated by DerSimonian and Laird method.

Supplementary Figure 16. ICU length of stay, without Mokhtar et al. 2025

Study or Subgroup	HFNC Mean	HFNC SD	HFNC Total	NIV Mean	NIV SD	NIV Total	Weight	Mean difference IV, Random, 95% CI
**1.4.1 General high risk subgroup, below 4 risk factors**								
✓ Hernandez et al 2016	3.75	1.4506	290	4.75	2.0259	314	18.0%	-1.00 [-1.28 , -0.72]
✕ Mokhtar et al 2025	16	1.744	60	17.5	2.2913	60	0.0%	-1.50 [-2.23 , -0.77]
**Subtotal**			**290**			**314**	**18.0%**	**-1.00 [-1.28 , -0.72]**
Test for overall effect: Z = 7.01 (P < 0.00001)								
Heterogeneity: Not applicable								
**1.4.2 Acute exarcebations of COPD with hypercapnic respiratory failure**								
✓ Jing et al 2019	8.5	3.5	22	9.4	4.8	20	8.7%	-0.90 [-3.46 , 1.66]
✓ RENOVATE 2025	3.25	1.5478	32	4.25	1.5207	39	16.8%	-1.00 [-1.72 , -0.28]
✓ Tan et al 2020	7.75	1.1815	44	8.825	1.8358	42	17.0%	-1.07 [-1.73 , -0.42]
**Subtotal (Wald[a])**			**98**			**101**	**42.5%**	**-1.04 [-1.51 , -0.56]**
Test for overall effect: Z = 4.27 (P < 0.0001)								
Heterogeneity: Tau² (DL[b]) = 0.00; Chi² = 0.03, df = 2 (P = 0.98); I² = 0%								
**1.4.3 Very high risk subgroup, 4 and above risk factors**								
✓ Hernandez et al 2022	12.675	3.5566	90	9.5	3.182	92	15.7%	3.18 [2.19 , 4.16]
✓ Tseng et al 2023	18	10	20	17	8.9	20	2.7%	1.00 [-4.87 , 6.87]
**Subtotal (Wald[a])**			**110**			**112**	**18.4%**	**3.12 [2.15 , 4.08]**
Test for overall effect: Z = 6.31 (P < 0.00001)								
Heterogeneity: Tau² (DL[b]) = 0.00; Chi² = 0.51, df = 1 (P = 0.47); I² = 0%								
**1.4.4 Non-operative obese subgroup**								
✓ Hernandez et al 2025	8.25	4.0543	72	12	16.4583	72	5.1%	-3.75 [-7.67 , 0.17]
**Subtotal**			**72**			**72**	**5.1%**	**-3.75 [-7.67 , 0.17]**
Test for overall effect: Z = 1.88 (P = 0.06)								
Heterogeneity: Not applicable								
**1.4.5 APACHE II score above 12 at extubation**								
✓ Shang et al 2021	6.88375	1.711667	24	7.19	1.585416	24	15.9%	-0.31 [-1.24 , 0.63]
**Subtotal**			**24**			**24**	**15.9%**	**-0.31 [-1.24 , 0.63]**
Test for overall effect: Z = 0.64 (P = 0.52)								
Heterogeneity: Not applicable								
**Total (Wald[a])**			**594**			**623**	**100.0%**	**-0.32 [-1.37 , 0.72]**

Test for overall effect: Z = 0.61 (P = 0.54)
Test for subgroup differences: Chi² = 68.89, df = 4 (P < 0.00001), I² = 94.2%
Heterogeneity: Tau² (DL[b]) = 1.55; Chi² = 69.44, df = 7 (P < 0.00001); I² = 90%

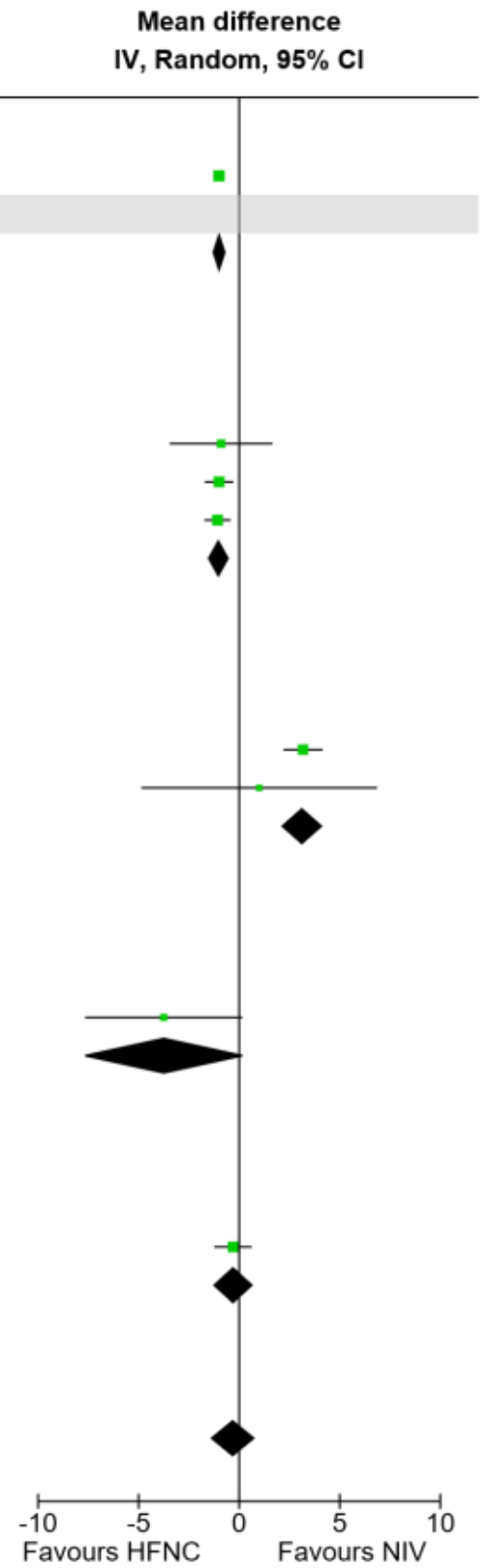

Footnotes
[a]CI calculated by Wald-type method.
[b]Tau² calculated by DerSimonian and Laird method.

<u>Publication bias assessment by Egger's test and Funnel Plots:</u>

Randomized Controlled Trials

Supplementary Figure 17. Reintubation within 72 hours.

Eggers test: z = -0.2457, p = 0.8060

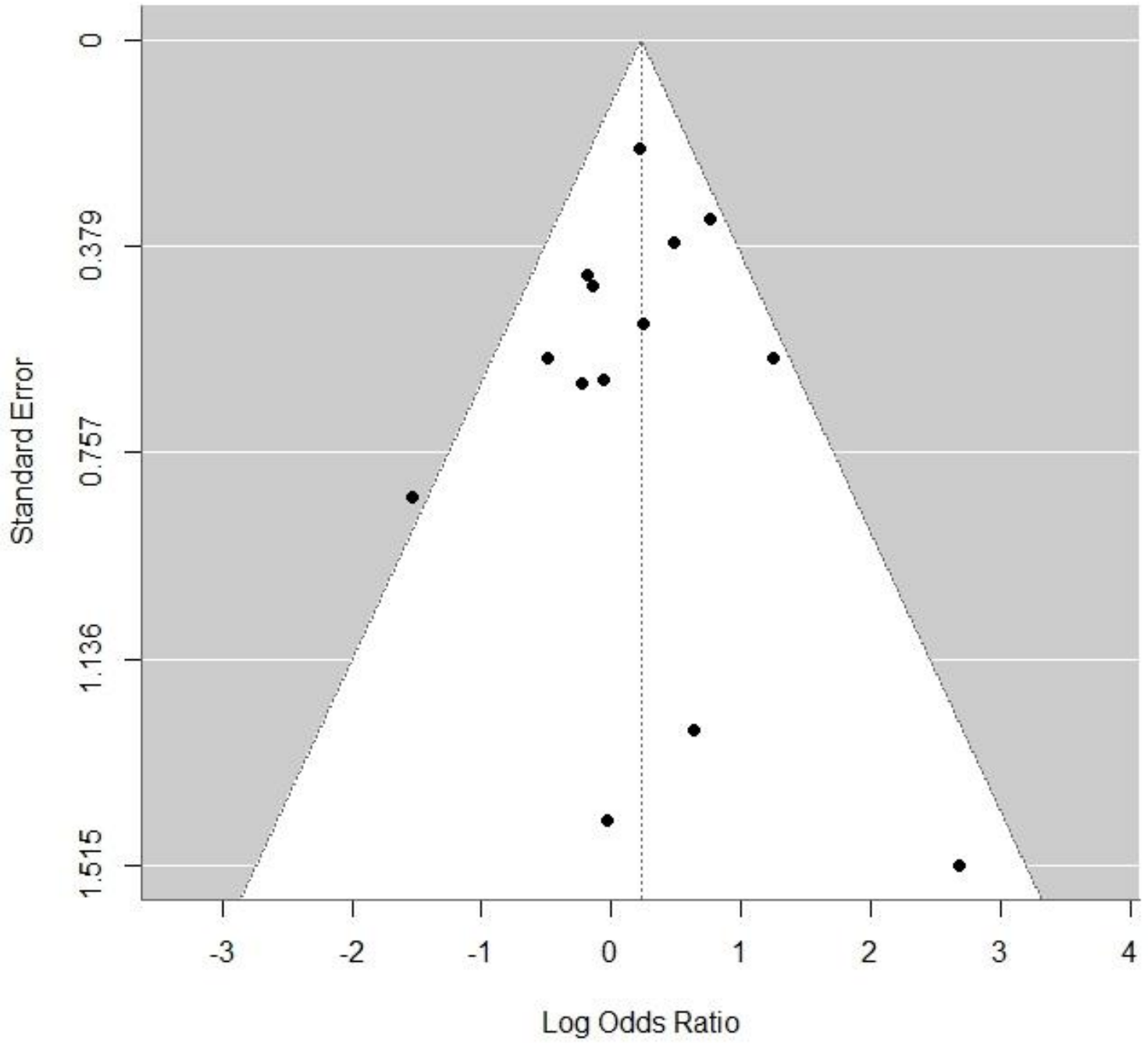

Supplementary Figure 18. ICU Mortality (no visual asymmetry in funnel plot)

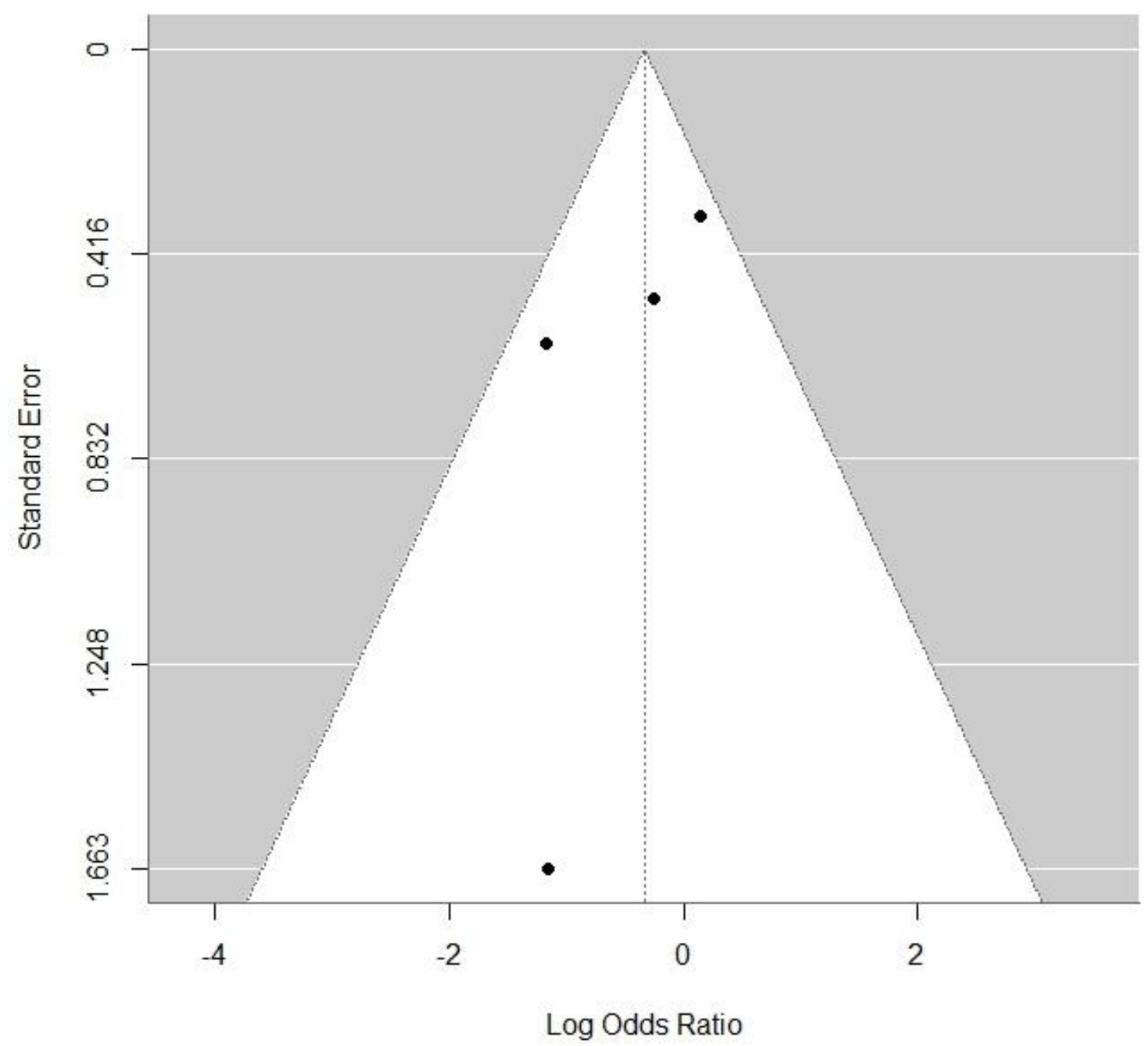

Supplementary Figure 19. Sepsis (no visual asymmetry in funnel plot)

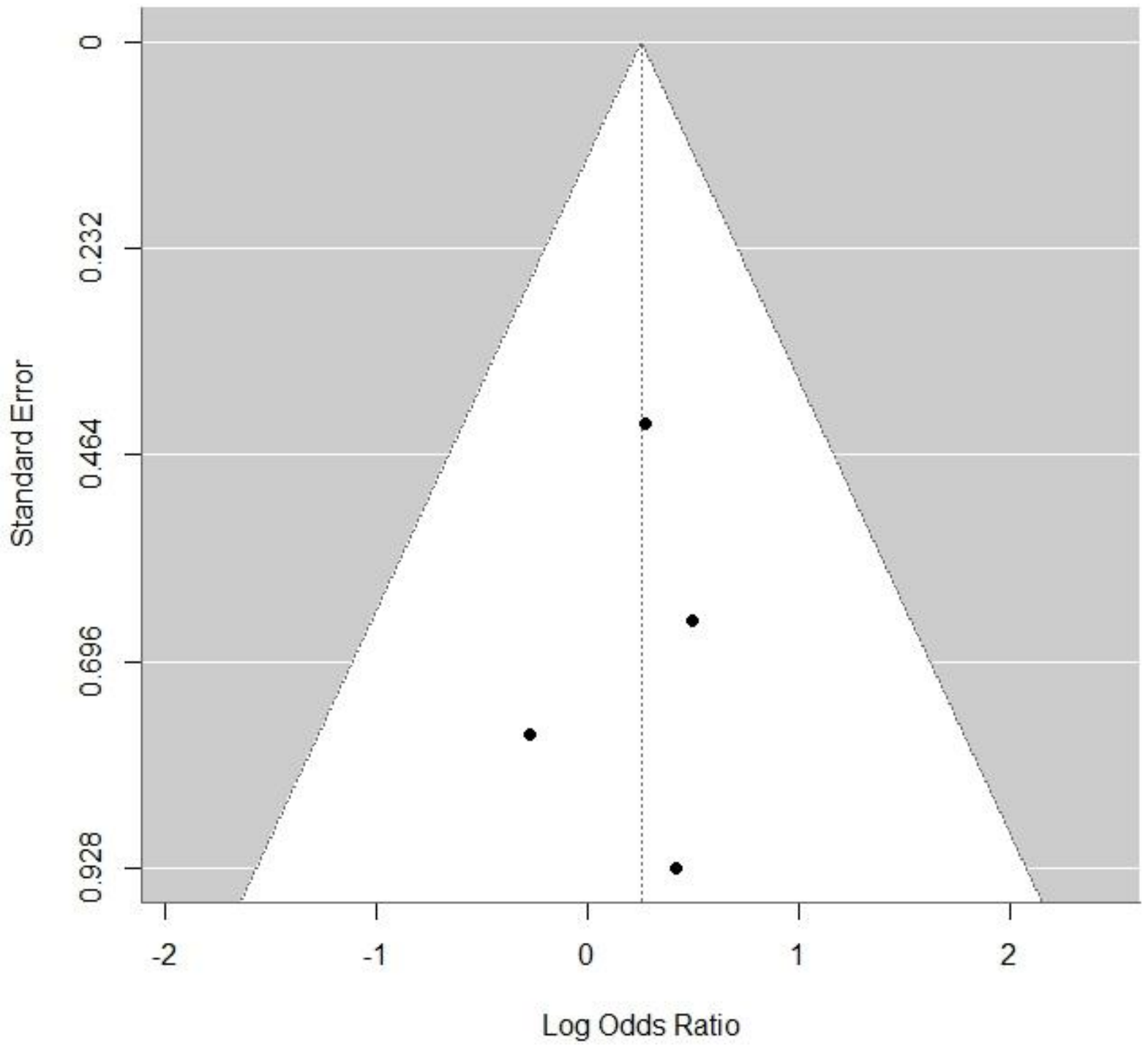

Supplementary Figure 20. Nosocomial Pneumonia (no visual asymmetry in funnel plot)

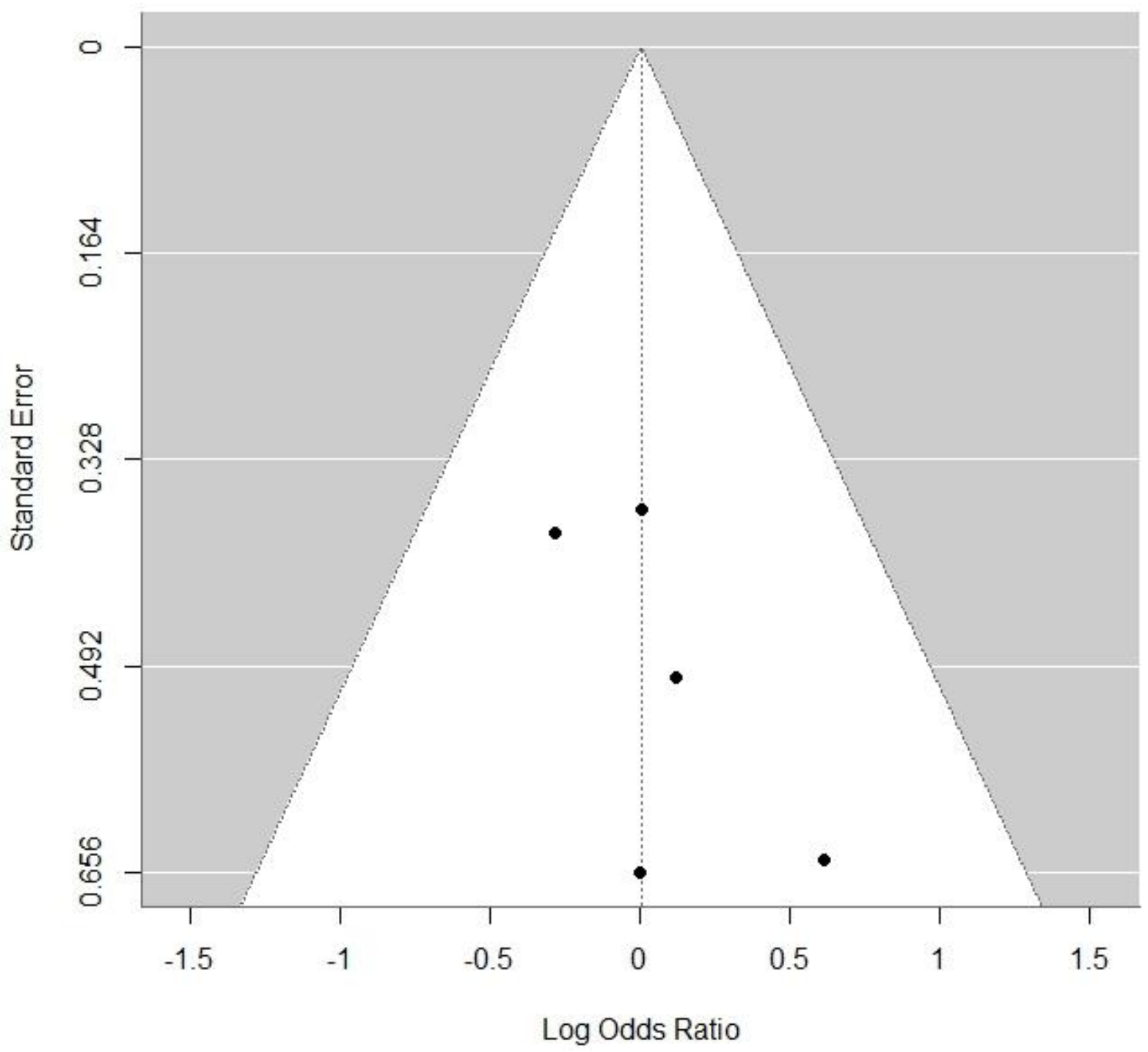

Supplementary Figure 21. ICU length of stay (no visual asymmetry in funnel plot)

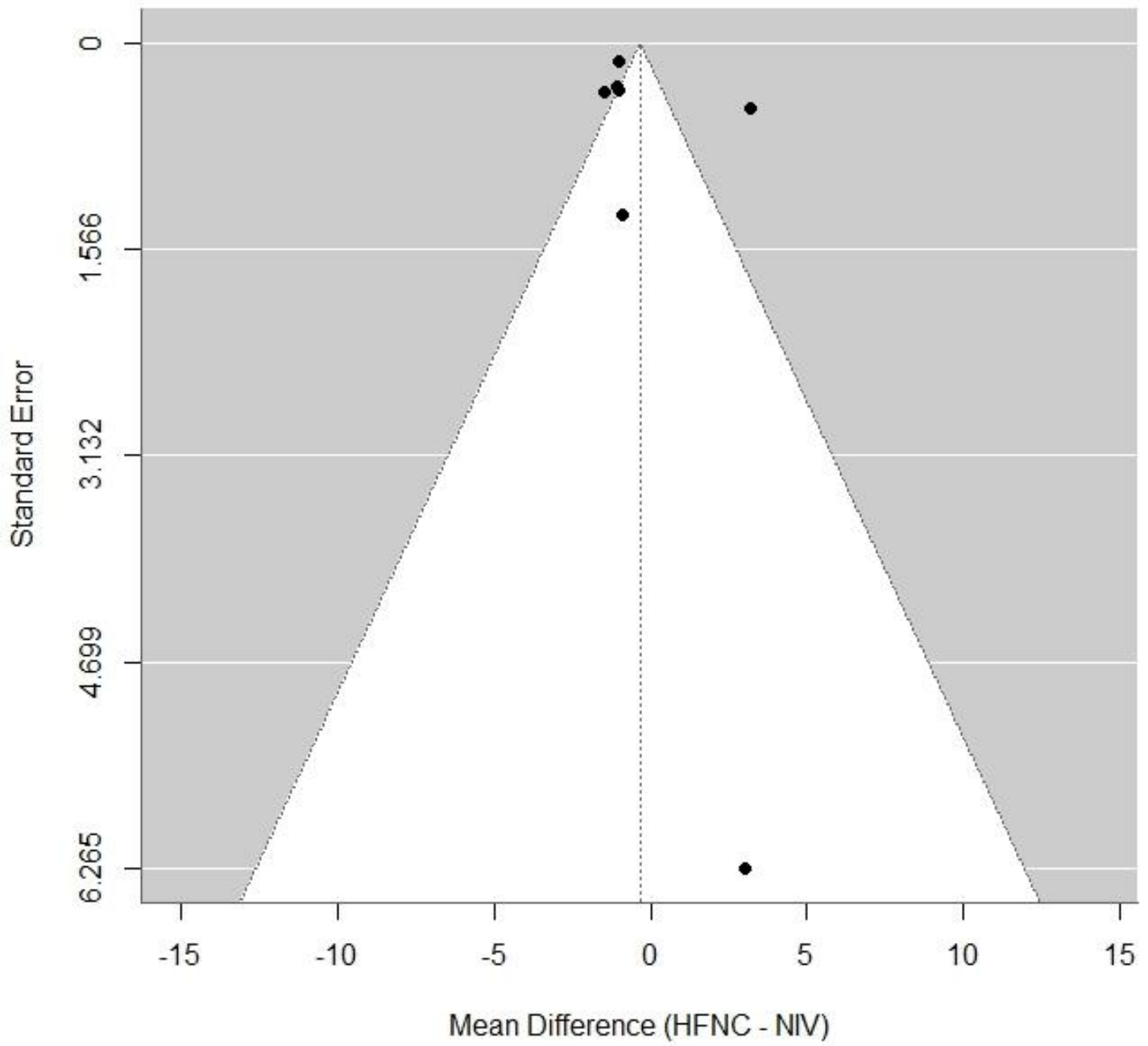

Subgroup analysis– Reintubation outcome

Supplementary Figure 22. Very High Risk, 4 or above risk factors (no visual asymmetry in funnel plot)

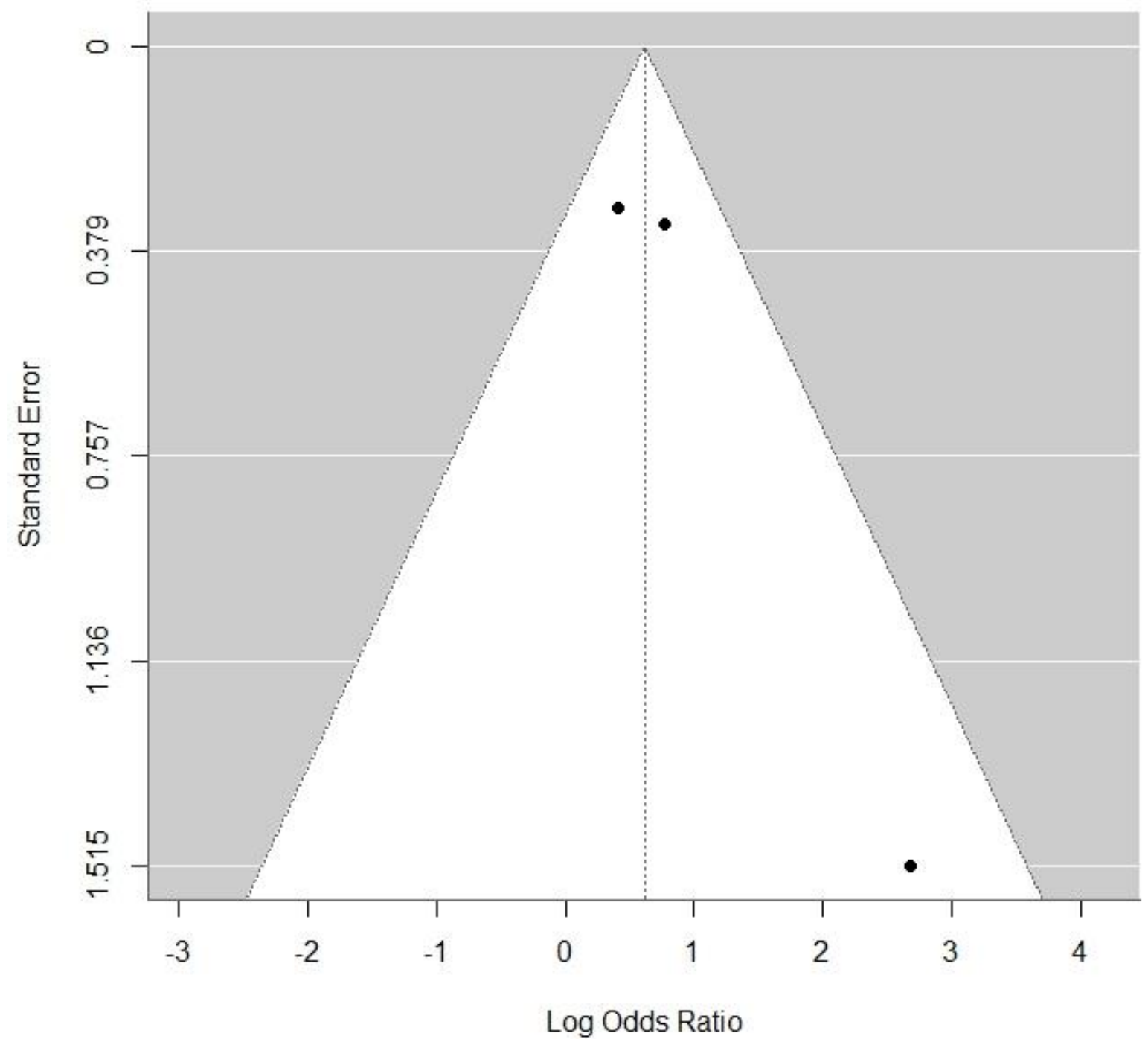

Supplementary Figure 23. General High Risk, below 4 risk factors

Eggers test: z = -0.8816, p = 0.3780

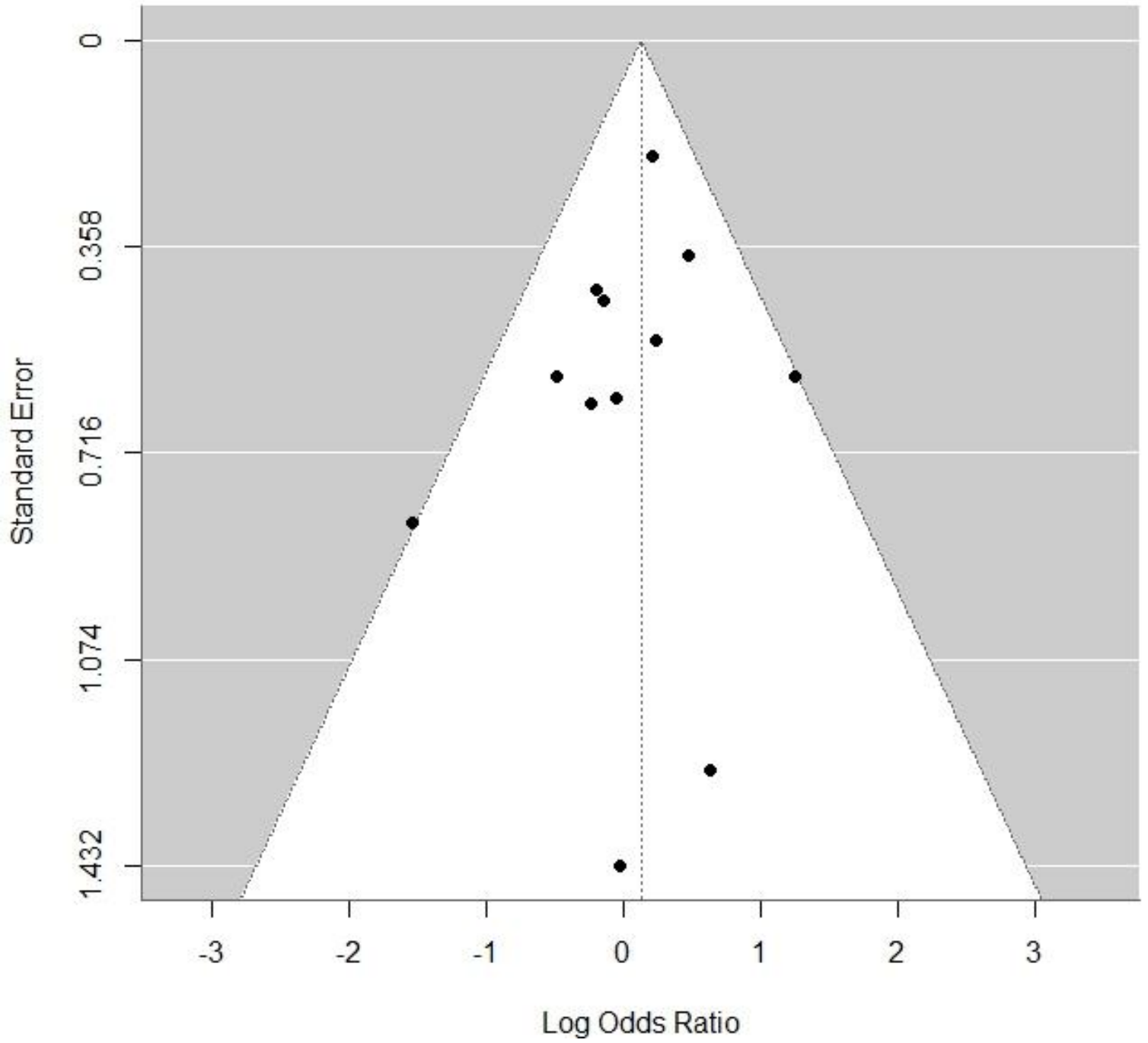

Supplementary Figure 24. Obese, INCLUDE Post-operative critically ill patients (no visual asymmetry in funnel plot)

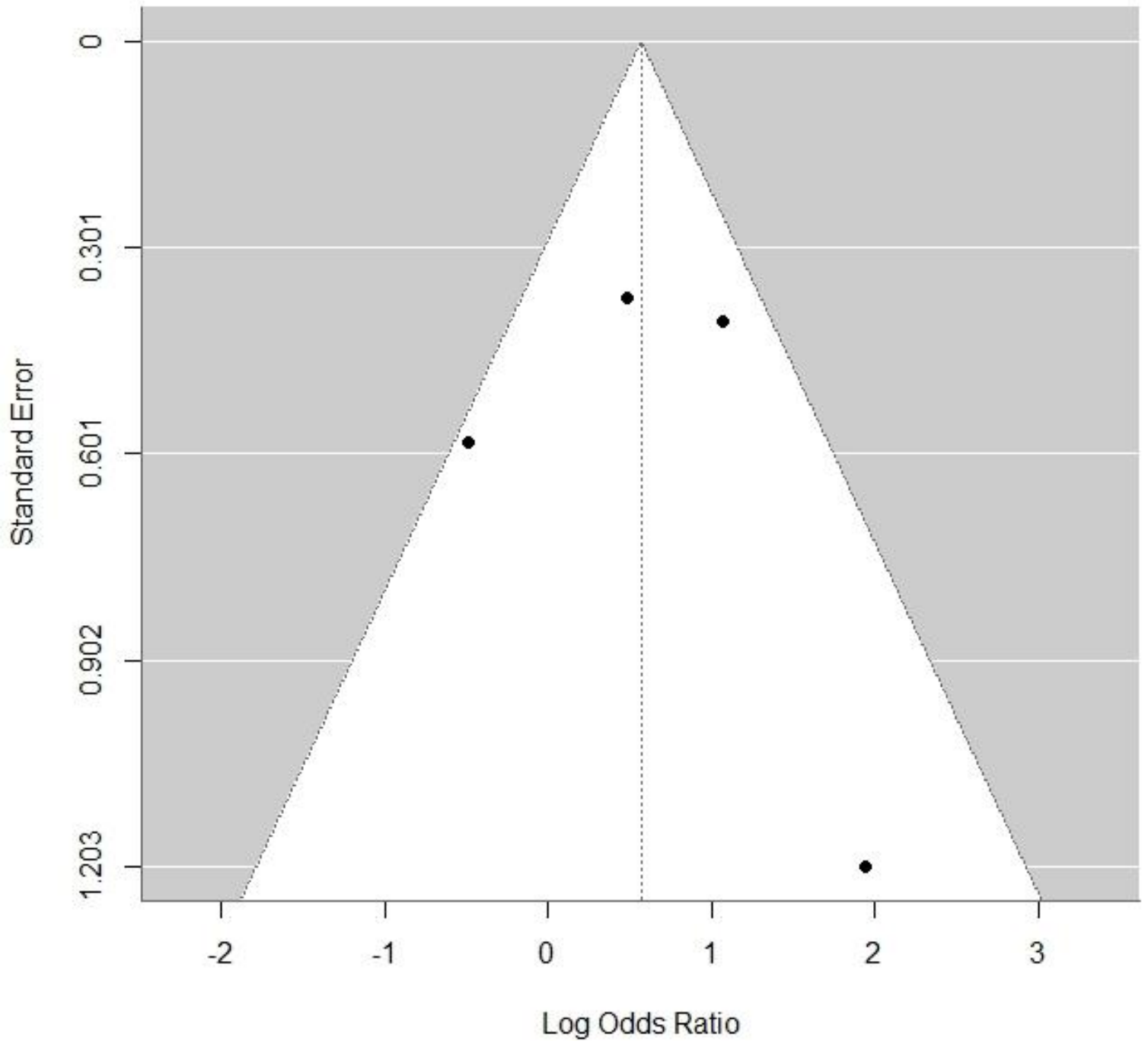

Supplementary Figure 25. Obese, EXCLUDE Post-operative critically ill patients (no visual asymmetry in funnel plot)

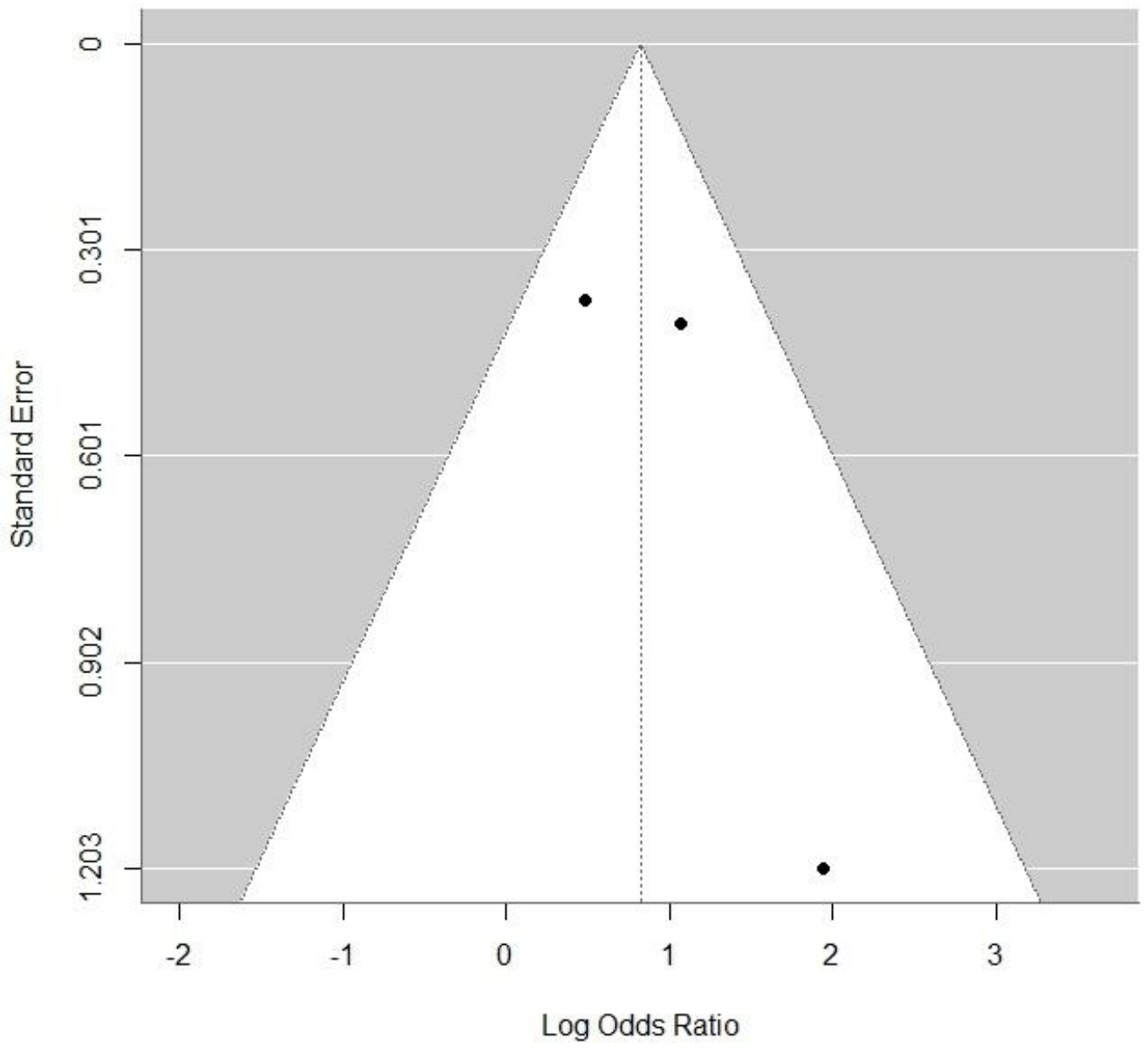

Supplementary Figure 26. Acute Exacerbation of COPD with hypercapnic respiratory failure (no visual asymmetry in funnel plot)

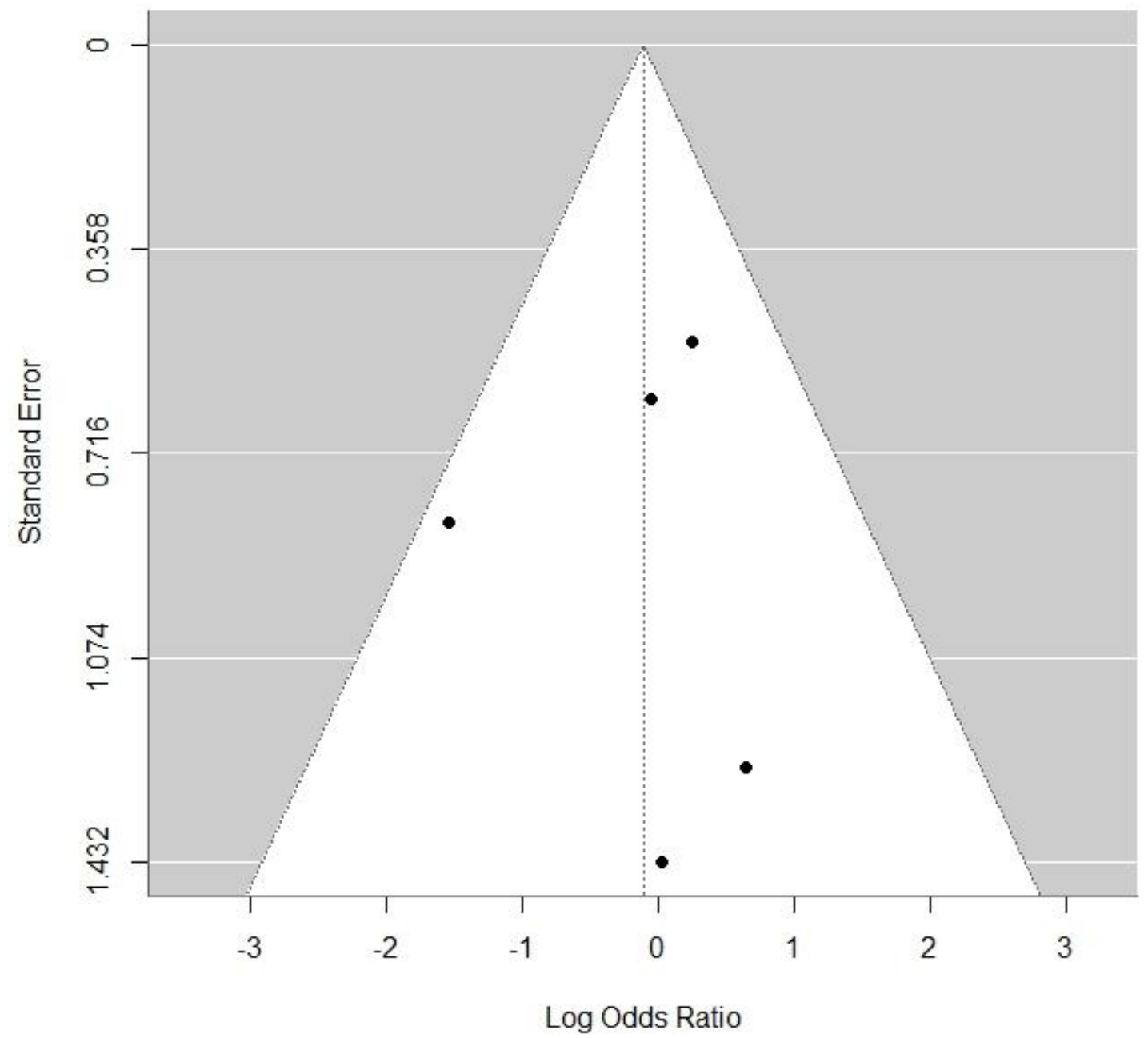

Figure 1. Flowchart of study selection.

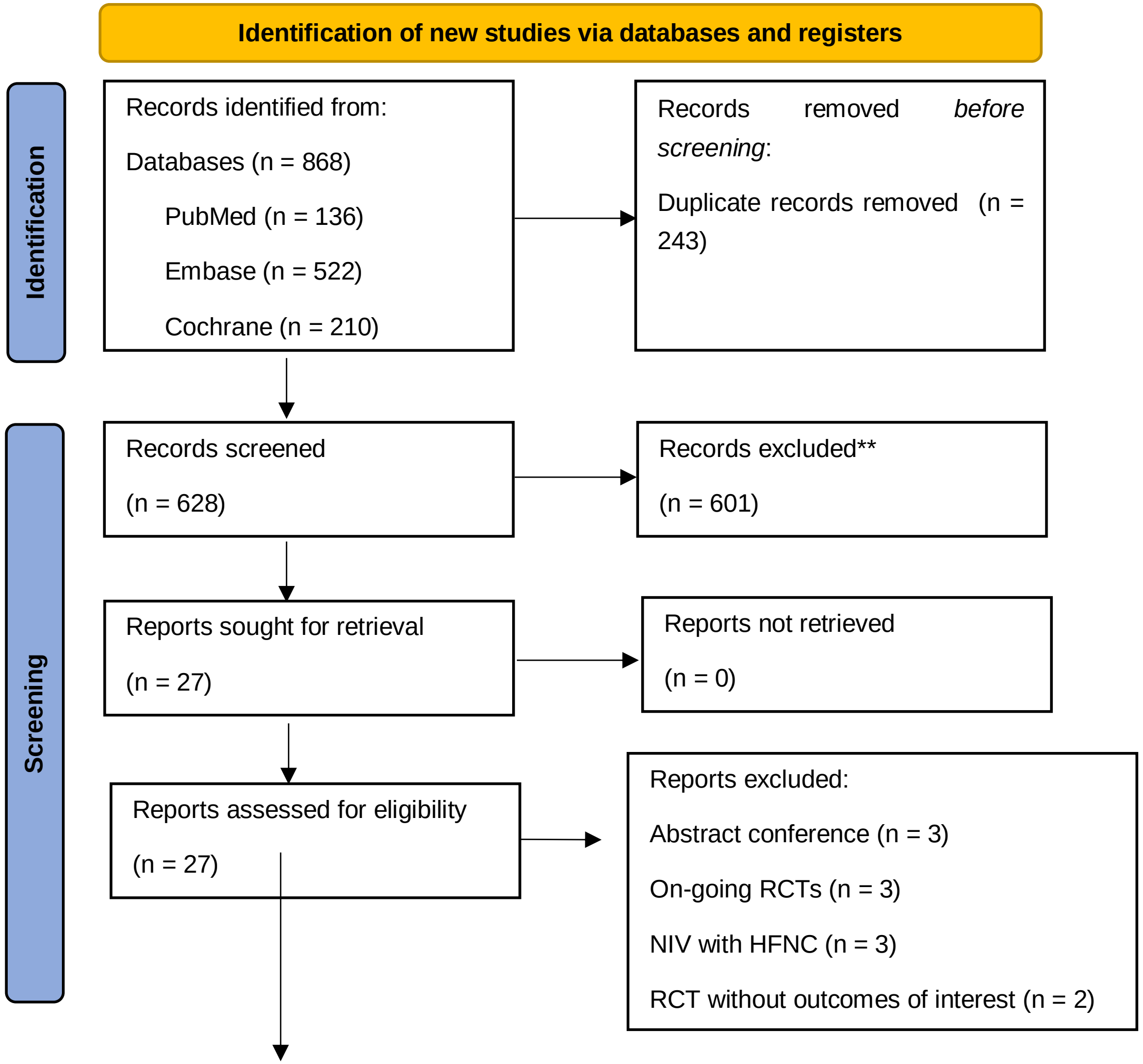

Included

Our main analysis:

- Total studies included in review (n = 16)
- Reports of total included studies (n = 16; 15 RCTs, with 1 post-hoc RCT analysis; 6 observational studies)

Supplementary Tab[illegible] Risk of bias summary (RoB 2)

Study	Bias from randomization process	Bias due to deviations from intended interventions	Bias due to missing outcome data	Bias in measurement of the outcomes	Bias in selection of the reported result	Overall risk of bias
Hernández et al. 2025	Low	Some concerns	Low	Low	Low	Some concerns
Hernández et al. 2016	Low	Some concerns	Low	Low	Low	Some concerns
Hernández et al. 2022	Low	Some concerns	Low	Low	Low	Some concerns
Tan et al. 2020	Low	Some concerns	Low	Low	Low	Some Concerns
Tongyoo et al. 2021	Low	Some concerns	Low	Low	Low	Some Concerns

Jing et al. 2019	Low	Some concerns	Low	Low	Low	Some Concerns
Tseng et al. 2023	Low	Some concerns	Low	Low	Low	Some Concerns
Mokhtar et al. 2025	Some concerns	Some concerns	Low	Low	Some concerns	High risk
Theerawit et al. 2021	Low	Some concerns	Low	Low	Low	Some Concerns
Cortegiani et al. 2020	Low	Some concerns	Low	Low	Low	Some concerns
Ketan et al. 2024	Some concerns	Some concerns	Low	Low	Low	High risk
Shang et al. 2020	Some concerns	Some concerns			Some concerns	High risk
RENOVATE 2025	Low	Some concerns	Low	Low	Low	Some concerns
Kumari et al. 2024	Low	Some concerns	Low	Low	Low	Some concerns

Stephan et al. 2017	Low	Some concerns	Low	Low	Low	Some concerns

Supplementary Table 2 summarizes the risk of bias assessments for the included studies.

Supplementary Table 3. All Excluded Trials

Study	**Reason of exclusion**
Thille et al 2018 [25]	Combined HFNC with NIV, compared to HFNC alone
Thille et al 2021 [26]	Post hoc analysis of the above study [24] focused on post-extubation respiratory failure
Bai et al. 2025 (HYPER-HOT NIV) [19]	On-going trials, and preliminary results published as conference abstract only
Temphattharachok et al. 2024 [20]	Conference abstract only
Thille et al 2021 [21]	Post hoc analysis of the [25] focused on COPD patients
De Jong et al 2025 [27]	Secondary analysis of the EXTUBOBESE trial, but did not provide subgroup analysi for HFNC alone in oxygen therapy group.
**NCT05918575 (RUSH UNIVERSITY) *[22]***	On-going trials, focusing on severely obese population.
NCT06671015 (YALE UNIVERSITY) [23]	On-going trials, focusing on systolic heart failure patients
Yang et al 2024 [24]	On-going trials, focusing on elderly with COPD
Liu et al 2023 [29]	Even though trial focused on AECOPD with type 2 respiratory failure, the outcomes were physiological. We acknowledged the reintubation rate but in

	% may impact the analysis heterogeneity. In stead, we mentioned this in the Discussion.
Arrive et al 2025 [28]	Trial focused on physiological outcomes of patients at high risk of reintubation

Section and Topic	Item #	Checklist item	Page where item is reported
**TITLE**			
Title	1	Identify the report as a systematic review.	1
**ABSTRACT**			
Abstract	2	See the PRISMA 2020 for Abstracts checklist.	3-4
**INTRODUCTION**			
Rationale	3	Describe the rationale for the review in the context of existing knowledge.	5
Objectives	4	Provide an explicit statement of the objective(s) or question(s) the review addresses.	5-6
**METHODS**			
Eligibility criteria	5	Specify the inclusion and exclusion criteria for the review and how studies were grouped for the syntheses.	7
Information sources	6	Specify all databases, registers, websites, organisations, reference lists and other sources searched or consulted to identify studies. Specify the date when each source was last searched or consulted.	8
Search strategy	7	Present the full search strategies for all databases, registers and websites, including any filters and limits used.	8
Selection process	8	Specify the methods used to decide whether a study met the inclusion criteria of the review, including how many reviewers screened each record and each report retrieved, whether they worked independently, and if applicable, details of automation tools used in the process.	8
Data collection process	9	Specify the methods used to collect data from reports, including how many reviewers collected data from each report, whether they worked independently, any processes for obtaining or confirming data from study investigators, and if applicable, details of automation tools used in the process.	8
Data items	10a	List and define all outcomes for which data were sought. Specify whether all results that were compatible with each outcome domain in each study were sought (e.g. for all measures, time points, analyses), and if not, the methods used to decide which results to collect.	9
	10b	List and define all other variables for which data were sought (e.g. participant and intervention characteristics, funding sources). Describe any assumptions made about any missing or unclear information.	N/A
Study risk of bias assessment	11	Specify the methods used to assess risk of bias in the included studies, including details of the tool(s) used, how many reviewers assessed each study and whether they worked independently, and if applicable, details of automation tools used in the process.	10
Effect measures	12	Specify for each outcome the effect measure(s) (e.g. risk ratio, mean difference) used in the synthesis or presentation of results.	9
Synthesis methods	13a	Describe the processes used to decide which studies were eligible for each synthesis (e.g. tabulating the study intervention characteristics and comparing against the planned groups for each synthesis (item #5)).	N/A
	13b	Describe any methods required to prepare the data for presentation or synthesis, such as handling of missing summary statistics, or data conversions.	N/A
	13c	Describe any methods used to tabulate or visually display results of individual studies and	N/A

		syntheses.	
	13d	Describe any methods used to synthesize results and provide a rationale for the choice(s). If meta-analysis was performed, describe the model(s), method(s) to identify the presence and extent of statistical heterogeneity, and software package(s) used.	10
	13e	Describe any methods used to explore possible causes of heterogeneity among study results (e.g. subgroup analysis, meta-regression).	10
	13f	Describe any sensitivity analyses conducted to assess robustness of the synthesized results.	10
Reporting bias assessment	14	Describe any methods used to assess risk of bias due to missing results in a synthesis (arising from reporting biases).	10
Certainty assessment	15	Describe any methods used to assess certainty (or confidence) in the body of evidence for an outcome.	10
**RESULTS**			
Study selection	16a	Describe the results of the search and selection process, from the number of records identified in the search to the number of studies included in the review, ideally using a flow diagram.	12
	16b	Cite studies that might appear to meet the inclusion criteria, but which were excluded, and explain why they were excluded.	12
Study characteristics	17	Cite each included study and present its characteristics.	12
Risk of bias in studies	18	Present assessments of risk of bias for each included study.	17
Results of individual studies	19	For all outcomes, present, for each study: (a) summary statistics for each group (where appropriate) and (b) an effect estimate and its precision (e.g. confidence/credible interval), ideally using structured tables or plots.	12-14
Results of syntheses	20a	For each synthesis, briefly summarise the characteristics and risk of bias among contributing studies.	N/A
	20b	Present results of all statistical syntheses conducted. If meta-analysis was done, present for each the summary estimate and its precision (e.g. confidence/credible interval) and measures of statistical heterogeneity. If comparing groups, describe the direction of the effect.	13-16
	20c	Present results of all investigations of possible causes of heterogeneity among study results.	17-18
	20d	Present results of all sensitivity analyses conducted to assess the robustness of the synthesized results.	18
Reporting biases	21	Present assessments of risk of bias due to missing results (arising from reporting biases) for each synthesis assessed.	18

Certainty of evidence	22	Present assessments of certainty (or confidence) in the body of evidence for each outcome assessed.	17-18
**DISCUSSION**			
Discussion	23a	Provide a general interpretation of the results in the context of other evidence.	19
	23b	Discuss any limitations of the evidence included in the review.	21
	23c	Discuss any limitations of the review processes used.	21
	23d	Discuss implications of the results for practice, policy, and future research.	20
**OTHER INFORMATION**			
Registration and protocol	24a	Provide registration information for the review, including register name and registration number, or state that the review was not registered.	2
	24b	Indicate where the review protocol can be accessed, or state that a protocol was not prepared.	N/A
	24c	Describe and explain any amendments to information provided at registration or in the protocol.	N/A
Support	25	Describe sources of financial or non-financial support for the review, and the role of the funders or sponsors in the review.	23
Competing interests	26	Declare any competing interests of review authors.	2
Availability of data, code and other materials	27	Report which of the following are publicly available and where they can be found: template data collection forms; data extracted from included studies; data used for all analyses; analytic code; any other materials used in the review.	23

Supplementary Table 4. PRISMA checklist

From: Page MJ, McKenzie JE, Bossuyt PM, Boutron I, Hoffmann TC, Mulrow CD, et al. The PRISMA 2020 statement: an updated guideline for reporting systematic reviews. BMJ 2021;372:n71. doi: 10.1136/bmj.n71. This work is licensed under CC BY 4.0. To view a copy of this license, visit https://creativecommons.org/licenses/by/4.0/

Supplementary Table 5. GRADE table

High-flow nasal cannula (HFNC) compared to non-invasive ventilation (NIV) for critically ill adults at high risk of reintubation
**Patient or population:** critically ill adults at high risk of reintubation<br>**Intervention:** high-flow nasal cannula (HFNC)<br>**Comparison:** non-invasive ventilation (NIV)

Outcomes	№ of participants (studies) Follow-up	Certainty of the evidence (GRADE)	Relative effect (95% CI)	Anticipated absolute effects	
				Risk with non-invasive ventilation (NIV)	Risk difference with high-flow nasal cannula (HFNC)
Reintubation within 72 hours	2073 (15 RCTs)	⊕⊕◯◯ Low[b,d]	**OR 1.22** (0.90 to 1.65)	165 per 1,000	**35 more per 1,000** (from 13 fewer to 84 more)
ICU Mortality	570 (4 RCTs)	⊕⊕◯◯ Low[b,d]	**OR 0.74** (0.40 to 1.38)	418 per 1,000	**71 fewer per 1,000** (from 195 fewer to 80 more)

High-flow nasal cannula (HFNC) compared to non-invasive ventilation (NIV) for critically ill adults at high risk of reintubation

Patient or population: critically ill adults at high risk of reintubation

Intervention: high-flow nasal cannula (HFNC)

Comparison: non-invasive ventilation (NIV)

Outcomes	№ of participants (studies) Follow-up	Certainty of the evidence (GRADE)	Relative effect (95% CI)	Anticipated absolute effects	
				Risk with non-invasive ventilation (NIV)	Risk difference with high-flow nasal cannula (HFNC)
Sepsis	1050 (4 RCTs)	⊕⊕◯◯ Low[b,d]	**OR 1.29** (0.71 to 2.35)	43 per 1,000	**12 more per 1,000** (from 12 fewer to 52 more)
Nosocomial Pneumonia	1321 (5 RCTs)	⊕⊕◯◯ Low[b,d]	**OR 1.01** (0.67 to 1.52)	77 per 1,000	**1 more per 1,000** (from 24 fewer to 36 more)
ICU length of stay	1145 (7 RCTs)	⊕◯◯◯ Very Low[a,b,d]	-		MD **0.37 day lower** (1.42 lower to 0.68 higher)

High-flow nasal cannula (HFNC) compared to non-invasive ventilation (NIV) for critically ill adults at high risk of reintubation

Patient or population: critically ill adults at high risk of reintubation

Intervention: high-flow nasal cannula (HFNC)

Comparison: non-invasive ventilation (NIV)

Outcomes	№ of participants (studies) Follow-up	Certainty of the evidence (GRADE)	Relative effect (95% CI)	Anticipated absolute effects	
				Risk with non-invasive ventilation (NIV)	Risk difference with high-flow nasal cannula (HFNC)
Reintubation within 72 hours (Exclusion of High Risk of Bias Studies, Ketan et al. 2024, Shang et al. 2021 and Mokhtar et al. 2025)	1843 (12 RCTs)	⨁⨁⨁◯ Moderate[d]	**OR 1.37** (1.05 to 1.77)	160 per 1,000	**47 more per 1,000** (from 7 more to 92 more)
Sepsis (Exclusion of High Risk of Bias Studies, Ketan et al. 2024, Shang et al. 2021 and Mokhtar et al. 2025)	930 (3 RCTs)	⨁⨁◯◯ Low[b,d]	**OR 1.26** (0.53 to 2.98)	21 per 1,000	**5 more per 1,000** (from 10 fewer to 39 more)
Nosocomial Pneumonia (Exclusion of High Risk of Bias Studies, Ketan et al. 2024, Shang et al. 2021 and Mokhtar et al. 2025)	1201 (4 RCTs)	⨁⨁◯◯ Low[b,d]	**OR 0.98** (0.63 to 1.54)	70 per 1,000	**1 fewer per 1,000** (from 25 fewer to 34 more)

High-flow nasal cannula (HFNC) compared to non-invasive ventilation (NIV) for critically ill adults at high risk of reintubation

Patient or population: critically ill adults at high risk of reintubation

Intervention: high-flow nasal cannula (HFNC)

Comparison: non-invasive ventilation (NIV)

Outcomes	№ of participants (studies) Follow-up	Certainty of the evidence (GRADE)	Relative effect (95% CI)	Anticipated absolute effects	
				Risk with non-invasive ventilation (NIV)	Risk difference with high-flow nasal cannula (HFNC)
ICU length of stay (Exclusion of High Risk of Bias Studies, Ketan et al. 2024, Shang et al. 2021 and Mokhtar et al. 2025)	1025 (6 RCTs)	⨁◯◯◯ Very Low[a,b,d]	-		MD **0.11 day lower** (1.39 lower to 1.17 higher)

*__The risk in the intervention group__ (and its 95% confidence interval) is based on the assumed risk in the comparison group and the **relative effect** of the intervention (and its 95% CI).

CI: confidence interval; **MD:** mean difference; **OR:** odds ratio

High-flow nasal cannula (HFNC) compared to non-invasive ventilation (NIV) for critically ill adults at high risk of reintubation

Patient or population: critically ill adults at high risk of reintubation

Intervention: high-flow nasal cannula (HFNC)

Comparison: non-invasive ventilation (NIV)

Outcomes	№ of participants (studies) Follow-up	Certainty of the evidence (GRADE)	Relative effect (95% CI)	Anticipated absolute effects	
				Risk with non-invasive ventilation (NIV)	Risk difference with high-flow nasal cannula (HFNC)

GRADE Working Group grades of evidence

High certainty: we are very confident that the true effect lies close to that of the estimate of the effect.

Moderate certainty: we are moderately confident in the effect estimate: the true effect is likely to be close to the estimate of the effect, but there is a possibility that it is substantially different.

Low certainty: our confidence in the effect estimate is limited: the true effect may be substantially different from the estimate of the effect.

Very low certainty: we have very little confidence in the effect estimate: the true effect is likely to be substantially different from the estimate of effect.

Explanations

a. **Inconsistency** was judged by forest plot inspection, I^2, and Chi^2 rather than I^2 cut-offs

b. **Imprecision** was rated down when 95% CIs crossed narrow clinical thresholds of importance.

Downgrading for imprecision may be considered if the 95% CI of an OR crosses below 0.75 or above 1.25. For dichotomous outcomes, a two-level downgrade may be warranted when the ratio of the upper to lower CI boundary exceeds 2.5 for ORs or 3 for RRs, reflecting a wide interval (*).

c. **Publication bias** was assessed using Egger's test; if $p < 0.05$, certainty of evidence was downgraded for risk of publication bias.

d. **Risk of bias**: All studies downgraded for deviations from intended interventions due to open-label design, and inclusion of three high risk trials at risk of bias from randomization process

Subgroups of Risk factor -Reintubation outcome

Patient or population: Critically ill patients

Intervention: High Flow Oxygen Nasal Canula

Comparison: Non-invasive Ventilation

Outcomes	№ of participants (studies) Follow-up	Certainty of the evidence (GRADE)	Relative effect (95% CI)	Anticipated absolute effects	
				Risk with Non-invasive Ventilation	Risk difference with High Flow Oxygen Nasal Canula
Very high risk, risk factdors of 4 and above	430 (3 RCTs)	⊕⊕⊕⊕ High	**OR 1.90** (1.12 to 3.21)	233 per 1,000	**133 more per 1,000** (from 21 more to 261 more)
High risk, below 4 risk factors	1595 (14 RCTs)	⊕⊕◯◯ Low[b,d]	**OR 1.34** (0.69 to 1.34)	156 per 1,000	**42 more per 1,000** (from 43 fewer to 42 more)
Obese, INCLUDE Post-operative Critically ill patients (without Kumari et al. 2024)	590 (4 RCTs)	⊕⊕◯◯ Very Low[a,b,d]	**OR 1.77** (0.83 to 3.77)	125 per 1,000	**77 more per 1,000** (from 19 fewer to 225 more)

Subgroups of Risk factor -Reintubation outcome

Patient or population: Critically ill patients

Intervention: High Flow Oxygen Nasal Canula

Comparison: Non-invasive Ventilation

Outcomes	№ of participants (studies) Follow-up	Certainty of the evidence (GRADE)	Relative effect (95% CI)	Anticipated absolute effects	
				Risk with Non-invasive Ventilation	Risk difference with High Flow Oxygen Nasal Canula
Obese, EXCLUDE Post-operative critically ill patients (without Kumari et al. 2024)	319 (3 RCTs)	⨁⨁⨁◯ Moderate[d]	**OR 2.26** (1.31 to 3.89)	180 per 1,000	**152 more per 1,000** (from 43 more to 281 more)
AECOPD with hypercapinc respiratory failure	346 (5 RCTs)	⨁⨁◯◯ Low[b,d]	**OR 0.89** (0.46 to 1.73)	148 per 1,000	**14 fewer per 1,000** (from 74 fewer to 83 more)

***The risk in the intervention group** (and its 95% confidence interval) is based on the assumed risk in the comparison group and the **relative effect** of the intervention (and its 95% CI).

CI: confidence interval; **OR:** odds ratio

Subgroups of Risk factor -Reintubation outcome

Patient or population: Critically ill patients

Intervention: High Flow Oxygen Nasal Canula

Comparison: Non-invasive Ventilation

Outcomes	№ of participants (studies) Follow-up	Certainty of the evidence (GRADE)	Relative effect (95% CI)	Anticipated absolute effects	
				Risk with Non-invasive Ventilation	Risk difference with High Flow Oxygen Nasal Canula

GRADE Working Group grades of evidence

High certainty: we are very confident that the true effect lies close to that of the estimate of the effect.

Moderate certainty: we are moderately confident in the effect estimate: the true effect is likely to be close to the estimate of the effect, but there is a possibility that it is substantially different.

Low certainty: our confidence in the effect estimate is limited: the true effect may be substantially different from the estimate of the effect.

Very low certainty: we have very little confidence in the effect estimate: the true effect is likely to be substantially different from the estimate of effect.

Explanations

a. **Inconsistency** was judged by forest plot inspection, I^2, and Chi^2 rather than I^2 cut-offs

b. **Imprecision** was rated down when 95% CIs crossed narrow clinical thresholds of importance.

Downgrading for imprecision may be considered if the 95% CI of an OR crosses below 0.75 or above 1.25. For dichotomous outcomes, a two-level downgrade may be warranted when the ratio of the upper to lower CI boundary exceeds 2.5 for ORs or 3 for RRs, reflecting a wide interval (*).

c. **Publication bias** was assessed using Egger's test; if $p < 0.05$, certainty of evidence was downgraded for risk of publication bias.

d. **Risk of bias**: All studies downgraded for deviations from intended interventions due to open-label design, and inclusion of three high risk trials at risk of bias from randomization process

Supplementary Table 6. Intervention details (HFNC vs NIV)

Study	High Flow Nasal Canula (HFNC)	Non-invasive Ventilation (NIV)
Hernández et al. 2025	Prior to extubation, gas was administered at 37°C and 10 L/min. Immediately after extubation, flow was rapidly **increased to 30 L/min** and **then titrated upward in 5 L/min increments until patient discomfort occurred.** Temperature was adjusted based on tolerance, with a minimum acceptable setting of 31°C. The FiO_2 was regularly titrated to **maintain SpO_2 >92%. After 48 hours,** HFNC was discontinued and conventional oxygen therapy was provided if needed to maintain adequate oxygenation.	Patients in the non-invasive ventilation (NIV) group **received BiPAP** therapy (V60 or V60 Plus; Philips) via a size-fitted face mask (Fisher & Paykel Healthcare) **for 48 hours following extubation**. PEEP and inspiratory pressure support were adjusted to maintain a respiratory rate ≤26 breaths/min and adequate gas exchange (SpO_2 ≥92% and pH ≥7.35), **with FiO_2 titrated to maintain SpO_2 ≥92%.** Sedatives were not permitted to improve NIV tolerance.
Hernández et al. 2016	**HFNC** was applied immediately after extubation. The **initial flow was set at 10 L/min and gradually increased in 5 L/min steps until patient discomfort occurred**. Temperature was set at 37°C and FiO_2 was adjusted to **maintain SpO_2 >92%.** HFNC was continued for **24 hours** postextubation, after which patients were switched to conventional oxygen therapy if needed.	**NIV** was delivered continuously via a full-face mask immediately after extubation for a **scheduled period of 24 hours**. After this, NIV was withdrawn, and oxygen was administered via a Venturi mask. Pressure support and PEEP were adjusted to target a respiratory rate of 25/min and adequate gas exchange. In this study, the **PEEP (positive end-expiratory pressure) for NIV** was **adjusted individually** to achieve a target respiratory rate of 25 breaths per minute and adequate gas exchange **(SpO_2 ≥92% with arterial pH ≥7.35).**
Hernández et al. 2022	HFNC was applied immediately before and after extubation using a size-adjusted nasal cannula, **with flow titrated from 10 L/min up to 60 L/min** as tolerated and temperature set at 37 °C, with FiO_2	NIV was applied immediately after extubation using a size-fitted **facemask with active humidification**, in bilevel positive airway pressure (**BiPAP**) mode, with PEEP and inspiratory pressure adjusted to

	adjusted to maintain **$SpO_2 \geq 92\%$ for 48 h.**	target a respiratory rate <26 breaths/min**, tidal volume 6–8 mL/kg** predicted body weight, and **$SpO_2 \geq 92\%$, for 48 h.**
Tan et al. 2020	**HFNC** patients **received 50 L/min** flow via large-bore nasal prongs at 37 °C and 44 mg H_2O/L humidity, with FiO_2 adjusted to maintain **SpO_2 88–92%.** Support lasted at least 2 h and continued as needed; interruptions were bridged with standard oxygen. Treatment success was defined as no need for respiratory support within **72 h** after discontinuation.	Patients randomized to **NIV** received S/T-mode ventilation via a full-face mask, **with initial EPAP of 4 cmH_2O and IPAP of 8 cmH_2O**, titrated to achieve adequate tidal volume, **SpO_2 88–92%**, respiratory rate ≤28/min, and $PaCO_2$ 45–60 mmHg.
Tongyoo et al. 2021	The **initial flow rate was set at 30 L/min** with a temperature of 37 °C, and the fraction of inspired oxygen (FiO_2) was started at 40% and adjusted to **maintain oxygen saturation ≥92%.** The flow rate was increased by 5 L/min every 10 minutes to meet patient demand, with a **maximum flow of 50 L/min**. HFNC was applied **continuously for 24 hours** and then gradually reduced based on the patient's clinical stability.	Drager Carina ventilator with a full facemask was used in bilevel positive airway pressure (**BiPAP**) mode. The initial inspiratory positive pressure (Pinsp) was 8 cmH_2O and expiratory positive pressure (Pexp) was 5 cmH_2O. **FiO_2 was started at 40% and adjusted to maintain oxygen saturation ≥92%.** Pinsp was increased by 2 cmH_2O every 10 minutes as needed, up to a maximum of 20 cmH_2O. NIV was applied **continuously for 24 hours**, except during airway secretion clearance, and the duration and settings were gradually reduced based on patient stability.
Jing et al. 2019	For HFNC, patients received oxygen via Optiflow or AIRVO™ 2 devices (Fisher & Paykel Healthcare, New Zealand) using size-appropriate nasal cannulas (<50% of nostril diameter), with humidifier temperature set at 37°C and **FiO_2 titrated to maintain SpO_2 between 88–92%.** Both HFNC and NIV were applied for at least 8 hours per day during the **first 48 hours post-extubation**.	For NIV, patients were supported with a VPAP III ST ventilator (ResMed, Australia) using a standard oronasal mask, with **inspiratory positive airway pressure initiated at 10–12 cmH_2O and expiratory positive airway pressure at 4–5 cmH_2O**, adjusted according to arterial blood gases, and **FiO_2 titrated to maintain SpO_2 88–92%.** Both HFNC and NIV were applied for at least 8 hours per

		day during **the first 48 hours post-extubation**.
Tseng et al. 2023	Patients assigned to the high-flow nasal cannula (HFNC) group received oxygen through an Optiflow system connected to the Airvo2 device. The initial **flow rate was set at 50 L/min**, and the gas temperature was adjusted between 34 and 37 °C according to the patient's airway secretion condition. Oxygen delivery was titrated to maintain adequate gas exchange, providing warmed, humidified oxygen with some positive airway pressure.	Patients in the noninvasive positive-pressure ventilation (NIPPV) group received ventilation using **a Trilogy 202 ventilator** with a facemask. **The inspiratory pressure was set between 12 and 16 cmH2O, and the end-expiratory pressure (PEEP) was set between 4 and 6 cmH2O** to maintain **a tidal volume of 6–10 mL/kg.** NIPPV provided controlled positive airway pressure to support ventilation, improve oxygenation, and reduce the risk of reintubation.
Mokhtar et al. 2025	In the HFNC group, a size-adjusted nasal cannula connected to the Bellavista™ 1000 ventilator was applied and delivered **continuously for 48 hours**, with an **initial flow of 10 L/min increased up to 60 L/min** according to patient comfort, and temperature maintained at 37°C. In both groups, FiO_2 was titrated to **maintain SpO_2 ≥92%,** and after 48 hours, patients were transitioned to conventional oxygen therapy as clinically indicated	In the NIV group, ventilatory support was provided continuously **for 48 hours using the GE™ CARESCAPE R860 ventilator** with a properly fitted oronasal mask, with PEEP and pressure support adjusted to maintain respiratory rate <26 breaths/min, **tidal volume 6–8 mL/kg** predicted body weight, **SpO_2 ≥92%,** and pH between 7.35–7.45. In both groups, FiO_2 was titrated to maintain SpO_2 ≥92%, and after 48 hours, patients were transitioned to conventional oxygen therapy as clinically indicated
Theerawit et al. 2021	HFNC (Optiflow; Fisher & Paykel Healthcare, New Zealand) was **delivered at 50 L/min** with an initial FiO_2 of 0.4, titrated to **maintain SpO_2 >92%.**	**The WhisperFlow CPAP system** provided **PEEP of 5 cmH₂O,** with FiO_2 initially set at 0.4 and adjusted to **achieve SpO_2 levels comparable to the HFNC group.**
Cortegiani et al. 2020	Heated, humidified HFNC (Optiflow/MR850 or AIRVO™ 2) initially set at **60 L/min** and **37 °C, with FiO_2 adjusted to maintain SpO_2 >90%.** Flow or temperature could be down-titrated for comfort.	NIV via full-face or oro-nasal mask in **pressure support ventilation (PSV) mode**, **PEEP 3–5 cm H_2O**, inspiratory pressure titrated to achieve **tidal volume 6–8 mL/kg ideal body weight**, **FiO_2**

	Applied continuously for the study period.	**adjusted to SpO_2 >90%.** Applied continuously for the study period.
Ketan et al. 2024	Heated and humidified HFNC was used with an initial flow of 15 L/min, titrated upward in 5 L/min increments according to patient tolerance, **up to an average of about 44 L/min,** with the temperature set at 37 °C and FiO2 adjusted to **maintain SpO2 between 88–92%.** Duration: **Initially 24 h**, continued as needed; discontinued when <4 h/day support	Patients received spontaneous/timed mode through an oronasal mask, with **PEEP starting at 4 cm H2O** and inspiratory pressure at 8 cm H2O, gradually increased to achieve **a tidal volume of ~6 mL/kg** and respiratory rate ≤24–28/min, while **FiO2 was adjusted to maintain SpO2 between 88–92%.** Duration: **Up to 72 h**
Shang et al. 2020	Warmed, humidified oxygen delivered via nasal cannula **at 40 L/min**, 37°C, with adjustable FiO_2 to maintain **SpO_2 >90%, applied for 24 h post-extubation.**	Noninvasive ventilation via mask in S/T mode with **inspiratory pressure 10–12 cmH_2O**, **expiratory pressure 4–6 cmH_2O**, I:E ratio 1.5–2.0, pressure rise 0.5–1 s, **FiO_2 adjusted to SpO_2 >90%**, applied for **24 h** post-extubation.
RENOVATE 2025	HFNO (Airvo-2, Fisher & Paykel Healthcare) was delivered continuously with flow rates **starting at 30–45 L/min** depending on the group and titrated to a maximum tolerated flow, with **FiO_2 adjusted to maintain SpO_2 88–92%** in **COPD exacerbation** patients and 92–98% in others.	NIV was delivered via face mask with **inspiratory positive airway pressure of 12–16 cm H_2O** (12–14 cm H_2O for non-COPD groups), **expiratory positive airway pressure of 4–8 cm H_2O**, and FiO_2 titrated to maintain target SpO_2. **Tidal volume was targeted at 6–9 mL/kg of** ideal body weight. Daily NIV usage and pressure settings were weaned according to patient tolerance and clinical improvement, with patients considered weaned when FiO_2 ≤30% and pressures ≤6 cm H_2O.
Kumari et al. 2024	Patients in the HFNC group received continuous heated and humidified oxygen therapy for **at least 48 hours after extubation,** initiated at a flow rate of **50 L/min** with FiO_2 titrated to **maintain SpO_2 >92%** and a humidifier temperature set at 37°C.	Patients in the NIV (**BiPAP**) group received ventilatory support via full facemask for at least 4 hours initially and a minimum of 12 **hours during the first 48 hours,** with pressure support adjusted to achieve **tidal volumes of 6–8 mL/kg**, **PEEP set between 5–10 cmH_2O**, and FiO_2 titrated to maintain

		**SpO_2 >92%.** All patients were continuously monitored in the ICU, and arterial blood gases were assessed at 1 hour and every 12 hours up to 48 hours.
**SUMMARY**	Across studies, HFNC was delivered via **heated, humidified, size-adjusted nasal cannulas** with **initial flows of 10–50 L/min**, titrated upward based on patient comfort, and gas temperature set around **37 °C**. **FiO_2 was adjusted to maintain SpO_2 ≥92%**, with some studies allowing 88–92% for specific populations. HFNC was applied **immediately before and/or after extubation** for **24–48 hours** or as clinically indicated. Flow and temperature were **modified for tolerance**, and patients were transitioned to conventional oxygen therapy once stable.	Across studies, **NIV was delivered via full-face or oronasal masks** in **BiPAP, S/T, or pressure support modes**, immediately after extubation. **Inspiratory pressure** was typically **8–16 cmH_2O** and **PEEP 3–10 cmH_2O**, titrated to achieve a **tidal volume of 6–10 mL/kg predicted body weight**, **respiratory rate ≤26–28/min**, and **adequate oxygenation (SpO_2 ≥88–92%)**. **FiO_2 was adjusted** to maintain target SpO_2, and **active humidification** was used in some studies. NIV was applied **continuously or for scheduled periods (24–72 h)**, with gradual weaning based on patient stability and tolerance. **Sedatives were generally avoided**, and arterial blood gases were monitored to guide ventilator adjustments.
