# Supplementary material for: High-Flow Nasal Cannula Versus Noninvasive Ventilation for Prevention of Reintubation in High-Risk Critically Ill Patients (HIGH-FLOW OXY): An Informative Systematic Review and Meta-Analysis
Source: Crit Care Explor. 2026 Aug 6;8(8):e1452. doi: 10.1097/CCE.0000000000001452 (PMC13446969; doi:10.1097/CCE.0000000000001452)
Supplement: Supplementary file 2 [file cc9-8-e1452-s002.pdf]

High Flow Oxygen Nasal Canula versus Non-invasive Ventilation

in Critically Ill patients at High Risk of Reintubation (HIGH-FLOW OXY):

A Systematic Review and Frequentist-Bayesian Pairwised Meta-Analysis

<u>For Cochrane and PubMed search strategy:</u>

(“High Flow Nasal Cannula”[Mesh]

OR high-flow nasal cannula[tiab]

OR high flow nasal oxygen[tiab]

OR HFNO[tiab]

OR HFNC[tiab]

OR “heated humidified high flow”[tiab])

AND (“Noninvasive Ventilation”[Mesh]

OR noninvasive ventilation[tiab]

OR NIV[tiab]

OR “non invasive ventilation”[tiab]

OR “non invasive respiratory support”[tiab])

AND (“Extubation Failure”[Mesh]

OR extubation failure[tiab]

OR reintubation[tiab]

OR re-intubation[tiab]

OR postextubation[tiab]

OR “extubation outcome”[tiab])

AND (“Intensive Care Units”[Mesh]

OR ICU[tiab]

OR critically ill[tiab]

OR critical care[tiab]

OR “high risk”[tiab]

OR “high-risk”[tiab])

<u>EMBASE search strategy:</u>

('noninvasive ventilation'/exp OR 'noninvasive ventilation' OR 'non invasive ventilation':ti,ab OR niv:ti,ab OR nippv:ti,ab OR bipap:ti,ab OR cpap:ti,ab) AND ('high flow nasal cannula'/exp OR 'high flow nasal cannula' OR 'high flow nasal oxygen':ti,ab OR hfnc:ti,ab OR hfno:ti,ab OR 'high flow therapy':ti,ab) AND ('reintubation'/exp OR 'reintubation' OR reintubat*:ti,ab OR 'extubation failure':ti,ab OR postextubation:ti,ab OR 'post extubation':ti,ab) AND ('intensive care unit'/exp OR 'intensive care unit' OR icu:ti,ab OR 'critically ill':ti,ab OR 'critical care':ti,ab OR 'high risk':ti,ab OR 'high-risk':ti,ab)

(("Noninvasive Ventilation"[Mesh] OR "noninvasive ventilation"[tiab] OR NIV[tiab] OR NIPPV[tiab] OR BiPAP[tiab] OR CPAP[tiab])

AND

("High-Flow Nasal Cannula"[Mesh] OR "high-flow nasal oxygen"[tiab] OR HFNC[tiab] OR HFNO[tiab] OR "high flow therapy"[tiab]))

AND

("Reintubation"[Mesh] OR reintubat*[tiab] OR "extubation failure"[tiab] OR "post-extubation"[tiab]))

AND

(“Intensive Care Units”[Mesh] OR ICU[tiab] OR critically ill[tiab] OR critical care[tiab] OR “high risk”[tiab] OR “high-risk”[tiab]))
